# Recapitulating infection, thermal sensitivity and antiviral treatment of seasonal coronaviruses in human airway organoids

**DOI:** 10.1016/j.ebiom.2022.104132

**Published:** 2022-06-29

**Authors:** Pengfei Li, Yining Wang, Mart M. Lamers, Marla Lavrijsen, Cinta Iriondo, Annemarie C. de Vries, Robbert J. Rottier, Maikel P. Peppelenbosch, Bart L. Haagmans, Qiuwei Pan

**Affiliations:** a Department of Gastroenterology and Hepatology, Erasmus MC-University Medical Center, Rotterdam, the Netherlands; b Viroscience Department, Erasmus MC-University Medical Center, Rotterdam, the Netherlands; c Department of Pediatric Surgery, Erasmus MC-Sophia Children's Hospital, Rotterdam, the Netherlands; d Department of Cell Biology, Erasmus MC-University Medical Center, Rotterdam, the Netherlands

**Keywords:** Airway organoids, Seasonal coronaviruses, Thermal sensitivity, Virus-host interactions, Antiviral therapy, hAOs, human airway organoids, HCoVs, human coronaviruses, AEM, airway organoid expansion medium, CBM, complete base medium, TEER, Transepithelial electrical resistance, CPE, cytopathic effect, TCID50, Median Tissue Culture Infectious Dose, PI, Propidium iodide, PCA, Principal component analysis, ISGs, Interferon-stimulated genes, GO, gene ontology, dsRNA, double-stranded RNA, ACE2, angiotensin converting enzyme 2, IFN, type I interferon, ANPEP, aminopeptidase

## Abstract

**Background:**

Human seasonal coronaviruses usually cause mild upper-respiratory tract infection, but severe complications can occur in specific populations. Research into seasonal coronaviruses is limited and robust experimental models are largely lacking. This study aims to establish human airway organoids (hAOs)-based systems for seasonal coronavirus infection and to demonstrate their applications in studying virus-host interactions and therapeutic development.

**Methods:**

The infections of seasonal coronaviruses 229E, OC43 and NL63 in 3D cultured hAOs with undifferentiated or differentiated phenotypes were tested. The kinetics of virus replication and production was profiled at 33 °C and 37 °C. Genome-wide transcriptome analysis by RNA sequencing was performed in hAOs under various conditions. The antiviral activity of molnupiravir and remdesivir, two approved medications for treating COVID19, was tested.

**Findings:**

HAOs efficiently support the replication and infectious virus production of seasonal coronaviruses 229E, OC43 and NL63. Interestingly, seasonal coronaviruses replicate much more efficiently at 33 °C compared to 37 °C, resulting in over 10-fold higher levels of viral replication. Genome-wide transcriptomic analyses revealed distinct patterns of infection-triggered host responses at 33 °C compared to 37 °C temperature. Treatment of molnupiravir and remdesivir dose-dependently inhibited the replication of 229E, OC43 and NL63 in hAOs.

**Interpretation:**

HAOs are capable of modeling 229E, OC43 and NL63 infections. The intriguing finding that lower temperature resembling that in the upper respiratory tract favors viral replication may help to better understand the pathogenesis and transmissibility of seasonal coronaviruses. HAOs-based innovative models shall facilitate the research and therapeutic development against seasonal coronavirus infections.

**Funding:**

This research is supported by funding of a VIDI grant (No. 91719300) from the Netherlands Organization for Scientific Research and the Dutch Cancer Society Young Investigator Grant (10140) to Q.P., and the ZonMw COVID project (114025011) from the Netherlands Organization for Health Research and Development to R.R.

**Research in context**

**Evidence before this study**

Seasonal coronaviruses usually cause mild upper respiratory tract illness, which indicates a preferential infection at upper airways. Anatomically, the temperature of upper and lower respiratory tract is different, and whether this disparity can affect seasonal coronavirus replication is largely unknown. Seasonal coronaviruses can cause severe complications and even mortality in a subset of patients, but there is no specific treatment. Furthermore, there is lacking of robust experimental models. Human airway organoids (hAOs) have recently been explored for modeling SARS-CoV-2 infection, but have not been studied for seasonal coronaviruses.

**Added value of this study**

This study demonstrated that seasonal coronaviruses 229E, OC43 and NL63 can efficiently infect hAOs with both undifferentiated and differentiated phenotypes. Notably, the enhanced level of replication at 33 °C compared to 37 °C may explain why most of the seasonal coronavirus infections cause upper respiratory tract illness. The potent antiviral activity of molnupiravir and remdesivir (two approved medications for treating COVID-19) against 229E, OC43 and NL63 support the repurposing for treating severe seasonal coronavirus infections.

**Implications of all the available evidence**

HAOs-based innovative models shall help to advance the research and therapeutic development against seasonal coronavirus infections.

Alt-text: Unlabelled box

## Introduction

Coronaviruses comprise a large number of zoonotic RNA viruses. There are seven coronavirus species that are known to infect human and cause common cold to severe respiratory infections.^1^ In contrast to the three highly pathogenic members (SARS-CoV-1, MERS-CoV and SARS-CoV-2), the four seasonal human coronaviruses (HCoVs)—NL63, 229E, OC43 and HKU1—have been largely neglected.^2^ In fact, seasonal HCoVs are widely circulating among the global population, which annually contribute to nearly 5% of the billions of upper respiratory infections.^3^ In general, they only cause self-limiting or mild upper respiratory illnesses, but recent evidence suggests that severe acute respiratory infections and even fatality can occur in specific vulnerable populations.^4^

Human respiratory tracts are lined by multi-ciliated cells, mucus-producing secretory cells and other columnar epithelium, forming an essential barrier for protection against microbial invasion.^5^ The anatomical structure of the upper and lower respiratory tract as well as the moderate distance ingeniously alter the temperature, from about 33 °C at the beginning of the upper respiratory tract to a body temperature of 37 °C in the lung.^6^^,^^7^ This temperature gradient can orchestrate the susceptibility and pathogenesis of different respiratory viruses.^7^, ^8^, ^9^ It is intriguing to hypothesize whether the lower temperature of the upper airway essentially favors the infection of seasonal HCoVs.

Organoids are self-renewing 3D constructs generated from tissue-resident stem cells. They can recapitulate the *in vivo* architecture, functionality of multi-cell types and genetic signature of original tissues.^10^ Human airway organoids (hAOs) from bronchial tissues consist of multiple cell lineages, including basal cells, ciliated cells, secretory cells, and club cells.^11^ These features collectively bestow hAOs unique advantages to model a multitude of pulmonary diseases, especially respiratory infections such as Influenza and SARS-CoV-2.^12^, ^13^, ^14^ Given the overall lacking of knowledge and experimental models for studying seasonal HCoVs, this study aims to first establish hAOs-based 229E, OC43 and NL63 infection models. Using these innovative models, we aim to further study the respiratory tract-related temperature on viral infection kinetics and virus-host interactions, as well as to explore potential antiviral treatment.

## Methods

### Viruses and cell lines

Vero E6 and Huh7 cells were maintained in Dulbecco's modified Eagle's medium (DMEM, Lonza, Belgium) supplemented with 10% fetal calf serum (FCS) (Hyclone, Logan, USA), penicillin (100 IU/mL) and streptomycin (100 μg /mL). Monkey LLC-MK2 cells were cultured in minimal essential medium with Earle's salt (MEM; Gibco, Grand Island, USA) containing 10% (vol/vol) FCS, 1x nonessential amino acid (Sciencell, San Diego, California, USA), 200 mM L-Glutamine (Lonza, Verviers, Belgium), 100 IU/mL penicillin and 100 μg/mL streptomycin. These cell lines were validated by short tandem repeat typing methods performed by the Pathology Department at Erasmus MC, and mycoplasma negative status was confirmed by monthly testing (commercially performed by GATC Biotech, Konstanz, Germany). HCoV-NL63 virus stock was produced by inoculating the virus onto LLC-MK2 cells and cultured at 33 °C. HCoV-OC43 and 229E stock were produced by inoculating the virus onto huh7 cells and cultured at 33 °C.

### Human airway organoids

Adult lung tissues were obtained from residual, tumor-free, material obtained at lung resection surgery for lung cancer. The Medical Ethical Committee of the Erasmus MC Rotterdam granted permission for this study (METC 2012-512). The isolation of human bronchial airway organoids were performed as previously described.^14^ Airway organoids (hAOs) were cultured in airway organoid expansion medium (AEM) as undifferentiated status, based on advanced DMEM/F12 (Invitrogen), supplemented with 1% penicillin/streptomycin (Life Technologies), 1 M HEPES (Life Technologies), 200 mM Ultraglutamine (Life Technologies), 2% (vol/vol) of B27 (Gibco), 1.25 mM N-acetylcysteine (Sigma-Aldrich), 10 mM Nicotinamide (Sigma-Aldrich), 10% (vol/vol) of R-spondin-1 (conditioned medium), 10% (vol/vol) of Noggin (conditioned medium), 100 ng/ml FGF10 (Peprotech), 25 ng/ml FGF7 (Peprotech), 1 µM SB202190 (Tocris), 500 nM A83-01 (Tocris) and 10 µM Y27632 (Sigma-Aldrich). To differentiate airway organoids, culture medium was changed into complete base medium (CBM; Stemcell Pneumacult-ALI) supplemented with 10 µM DAPT (Tocris).

### Inoculation of airway organoids with coronaviruses

Undifferentiated or differentiated hAOs were first mechanically dissociated into lumen outside (outer) or sheared into small fragments, then inoculated with cell culture-derived 229E, OC43 or NL63 virus particles (MOI = 0.5) for 2 h at 33 °C. HAOs from one well of 48 well plate were randomly selected and digested into single cells by Triple Express, for counting cell numbers and calculating MOI. Infected organoids were then washed by PBS for 3 times to thoroughly remove unabsorbed virus. Finally, organoids were embedded in Matrigel, and maintained in AEM or CBM with 10 µM DAPT at either 33 °C or 37 °C with 5% CO_2_. Organoids and supernatants were separately harvested to quantify virus RNA copies at 1 h, 1 day, 3 day, 5 day, 7 day after inoculation. For TCID 50 assay, organoids and supernatant were collectively harvest and used to calculate TCID 50 in cell lines.

### Genome-wide RNA sequencing analysis

Total RNA of hAOs was isolated at 48 h post-inoculation of the viruses. The experimental groups were designed as follows: Undifferentiated hAOs cultured at 33 °C as mock; Undifferentiated hAOs with 229E inoculation cultured at 33 °C; Undifferentiated hAOs with OC43 inoculation cultured at 33 °C; Undifferentiated hAOs cultured at 37 °C as mock; Undifferentiated hAOs with 229E inoculation cultured at 37 °C; Undifferentiated hAOs with OC43 inoculation cultured at 37 °C; Differentiated hAOs cultured at 33 °C as mock; Differentiated hAOs with 229E inoculation cultured at 33 °C; Differentiated hAOs with OC43 inoculation cultured at 33 °C; Differentiated hAOs cultured at 37 °C as mock; Differentiated hAOs with 229E inoculation cultured at 37 °C; Differentiated hAOs with OC43 inoculation cultured at 37 °C.

The quality of RNA was measured by Bioanalyzer RNA 6000 Picochip as quality-control step, followed by RNA sequencing performed by Novogene with paired-end 150 bp (PE 150) sequencing strategy. The identification of differentially expressed genes is based on *P* < 0.05 and absolute values of log2FoldChange > 0. Data are publically available at https://doi.org/10.17026/dans-xsq-ps55.

### Transwell culture and virus inoculation

HAOs were dissected into single cells and seeded on semipermeable transwell inserts (Corning BV) that were pre-coated with collagen. The upper insert and lower compartment were supplemented with AEM for the first week then maintained in CBM supplemented with DAPT for another two weeks. For virus inoculation, seasonal coronaviruses were inoculated through apical sides for 2 h at 33 °C. The apical sides were then washed by PBS for 3 times to remove unabsorbed viruses. Finally, both apical and basolateral compartments were maintained in AEM: CBM at a 1:3 ratio at 33 °C with 5% CO_2_. Supernatant from apical and basolateral sides was harvested at 1 h, 1 day, 2 day and 3 day post-inoculation. The TEER of monolayers on transwell inserts was measured by using an epithelial voltohmmeter (EVOM,^2^ World Precision Instruments).

### TCID 50 calculation

Viruses from hAOs and culture supernatants were collectively harvested through three-times freezing and thawing. HCoVs titer were quantified by using a 50% Tissue Culture Infectious Dose (TCID50) assay. Briefly, ten-fold dilutions of HCoV-229E and HCoV-OC43 were inoculated onto Huh7 cells and Vero-E6 cells respectively that grown in a 96-well plate at 2000 cells/well. The plate was incubated at 33 °C for 5–10 days, and each well was examined by counting cytopathic effect (CPE). The TCID50 value was calculated by using the Reed–Muench method.

### Antiviral drug treatment

Undifferentiated hAOs were inoculated with 229E, OC43 or NL63 virus particles and cultured in 3D structure as described. Then hAOs were cultured in AEM supplemented with molnupiravir (MedChem Express, USA) or remdesivir (MedChem Express, USA) in serial concentrations for 48 h at 33 °C or 37 °C. After 48 h treatment, organoids were lysed for further analysis.

### AlamarBlue assay

Culture supernatant of hAOs was discarded and then organoids were incubated with Alamar Blue (Invitrogen, DAL1100, 1:20 dilution in AEM) for two hours (37 °C). Next, all the medium was collected for analyzing the metabolic activity of the organoids. Absorbance was determined by using fluorescence plate reader (CytoFluor Series 4000, Perseptive Biosystems) at the excitation of 530/25 nm and emission of 590/35 nm.

### Quantification of coronaviruses genome copy numbers

229E, OC43 or NL63 virus genome that contain qPCR detection region were used to generate plasmids. These plasmids were used as template for quantifying corresponding coronavirus genome copy number. A series of dilutions of plasmid from 10^−1^ to 10^−9^ were prepared, and then were amplified and quantified by qRT-PCR to generate a standard curve. Standard curve was generated by plotting the log copy number versus the cycle threshold (CT) value. Coronavirus copy numbers were calculated as following equation: Copy number (molecules/μl)= [concentration (ng/μl)x6.022 × 10^23^ (molecules/mol)]/[length of ampliconx660 (g/mol)x10^9^ (ng/g)].

### Detection of organoids cell death

The 229E or OC43 infected hAOs were cultured at 33 °C or 37 °C for 7 days. Next, hAOs were stained with propidium iodide (PI) (Sigma-Aldrich) and Calcein AM (Invitrogen). Healthy hAOs without infections were cultured at 33 °C or 37 °C as mock. Images were detected using Leica SP5 cell imaging system.

### Immunofluorescence assay

First, hAOs were washed in cold AdDMEM/F12 medium for 3 times to remove all basal matrix. Subsequently, hAOs were added into the CytoSpin II Cytocentrifuge (Shandon Scientifi Ltd, Runcorn, England) and spun down into slides at 800 rpm for 5 min. Next, hAOs were fixed in 4% paraformaldehyde solution for 15 min. The slides containing organoids were then rinsed 3 times with PBS, followed by permeabilizing with PBS containing 0.2% (vol/vol) tritonX100 for 10 min. Then the slides were twice rinsed with PBS for 5 min, followed by incubation with blocking solution (5% donkey serum, 1% bovine serum albumin, 0.2% tritonX100 in PBS) at room temperature for 1 h. Next, slides were incubated with primary antibody diluted in blocking solution at 4 °C overnight. Primary antibodies used in this study are as follows: anti-EpCAM antibody (1:1000, rabbit mAb; Abcam), anti-dsRNA antibody (1:500, mouse mAb; Scicons J2), anti-β-Tubulin antibody (1:500, rabbit mAb; Cell signaling), anti-MUC5AC antibody (1:500, rabbit mAb; Cell signaling), anti-Keratin 5 antibody (1:1000, rabbit mAb; Cell signaling), and anti-CC10 antibody (1:200, mouse mAb, Santa Cruz Biotechnology), human IVIg (1:50, Biotest). Slides were washed 3 times for 5 min each in PBS prior to 1 h incubation with 1:1000 dilutions of the anti-mouse IgG (H+L, Alexa Fluor® 594), the anti-rabbit IgG (H+L, Alexa Fluor® 488), the anti-human IgG (H+L, Alexa Fluor® 594) secondary antibodies. Nuclei were stained with DAPI (4, 6-diamidino-2-phenylindole; Invitrogen).

### Statistics

The statistical significance of differences between means was assessed with the Mann-Whitney test (GraphPad Prism; GraphPad Software Inc., La Jolla, CA). The threshold for statistical significance was defined as *P* ≤ 0.05.

### Regent validation

All chemical reagents and antibodies used in this study are commercially available. The antibodies have been validated in either immunofluorescence or immunohistochemical assays in multiple previous studies as showed in manufacturer's website.

### Role of Funders

Funders had no role in study design, data collection, data analyses, interpretation, or writing of report.

## Results

**Figure 1:**
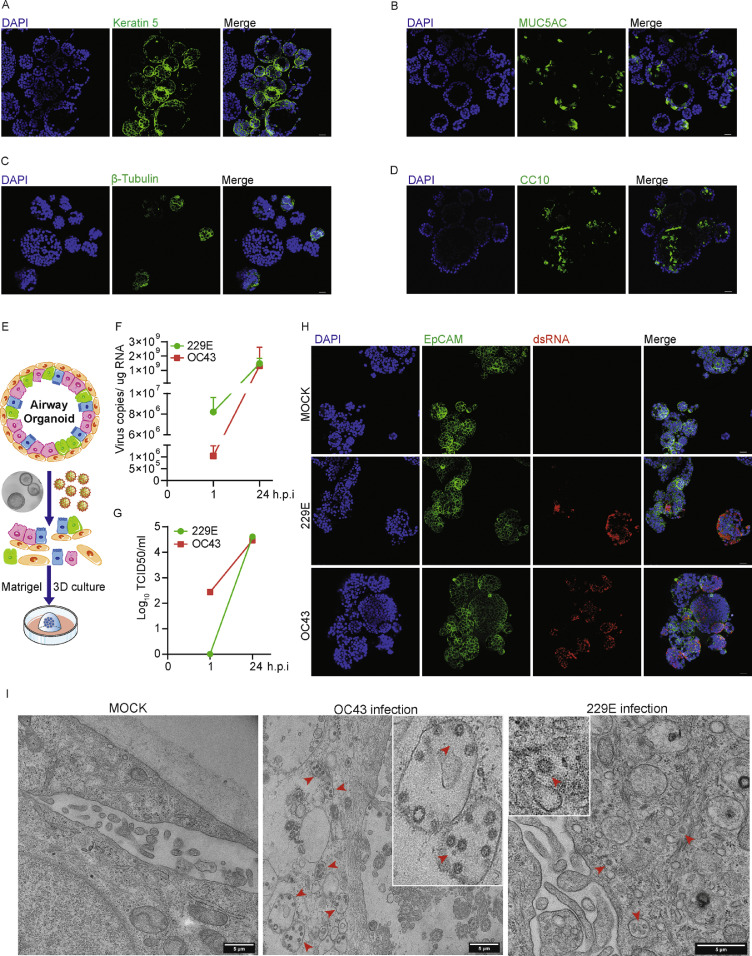
Characterizing the cell types of undifferentiated hAOs and the permission of hAOs to 229E and OC43 infections. (A) to (D) Characterizing cell types by immunostaining Kertain 5 (basal cells), MUC5AC (goblet cells), β-tubulin (ciliated cells) and CC10 (club cells). (E) schematic diagram for seasonal coronaviruses inoculation in hAOs. (F) Dynamics of intracellular virus RNA copies in hAOs cultured at 33 °C from 1 to 24 h post-inoculation (*n*=3). (G) Dynamics of infectious viral titers in hAOs cultured at 33 °C from 1 to 24 h post-inoculation (*n*=3). (H) Immunofluorescence staining for viral double-stranded RNA (dsRNA) in hAOs at 33 °C. Scale bar = 25 μm. (I) Transmission electron microscopy analysis of 229E and OC43 infected hAOs cultured with expansion medium at 33 °C.

To characterize the major cell types of 3D cultured hAOs, we immunostained specific cell markers Keratin 5 (basal cells), MUC5AC (goblet cells), β-Tubulin (ciliated cells) and CC10 (club cells). As shown, hAOs cultured with expansion medium (undifferentiated phenotype) were characterized by a major layer of basal cells, followed by goblet cells, club cells and ciliated cells (Figure 1A to D). These hAOs were inoculated with 229E and OC43 virus particles and cultured at 33 °C, a temperature resembling the upper respiratory tract (Figure 1E). Viral RNA levels were quantified by qRT-PCR and calculated as copy numbers (Supplementary Figure 1). As compared between 1 and 24 h post-inoculation, rapid growth of 229E was evidenced by dramatic increase of viral RNA levels from 8.2 × 10^6^ to 1.5 × 10^9^ copies/ug RNA and the infectious viral titers from 0 to 10^4.7^ TCID50/ml. Similar results were observed when inoculating hAOs with OC43 virus particles (Figure 1F and G). Robust viral replication was further demonstrated by immunostaining viral dsRNA, the replication intermediate (Figure 1H). Transmission electron microscopy analysis was performed at 48 hours after virus inoculation, and OC43 and 229E virus particles were visualized in organoid cells (Figure 1I). We next tested the susceptibility of hAOs to NL63 infection. Quantification of intracellular viral RNA copies indicated that NL63 can stably replicate in hAOs culturing at 33 °C as measured from 1 h to 7 days post-inoculation (Supplementary Figure 2A). Immunostaining further demonstrated viral replication in distal hAOs cultured at 33 °C (Supplementary Figure 2B).

**Figure 2:**
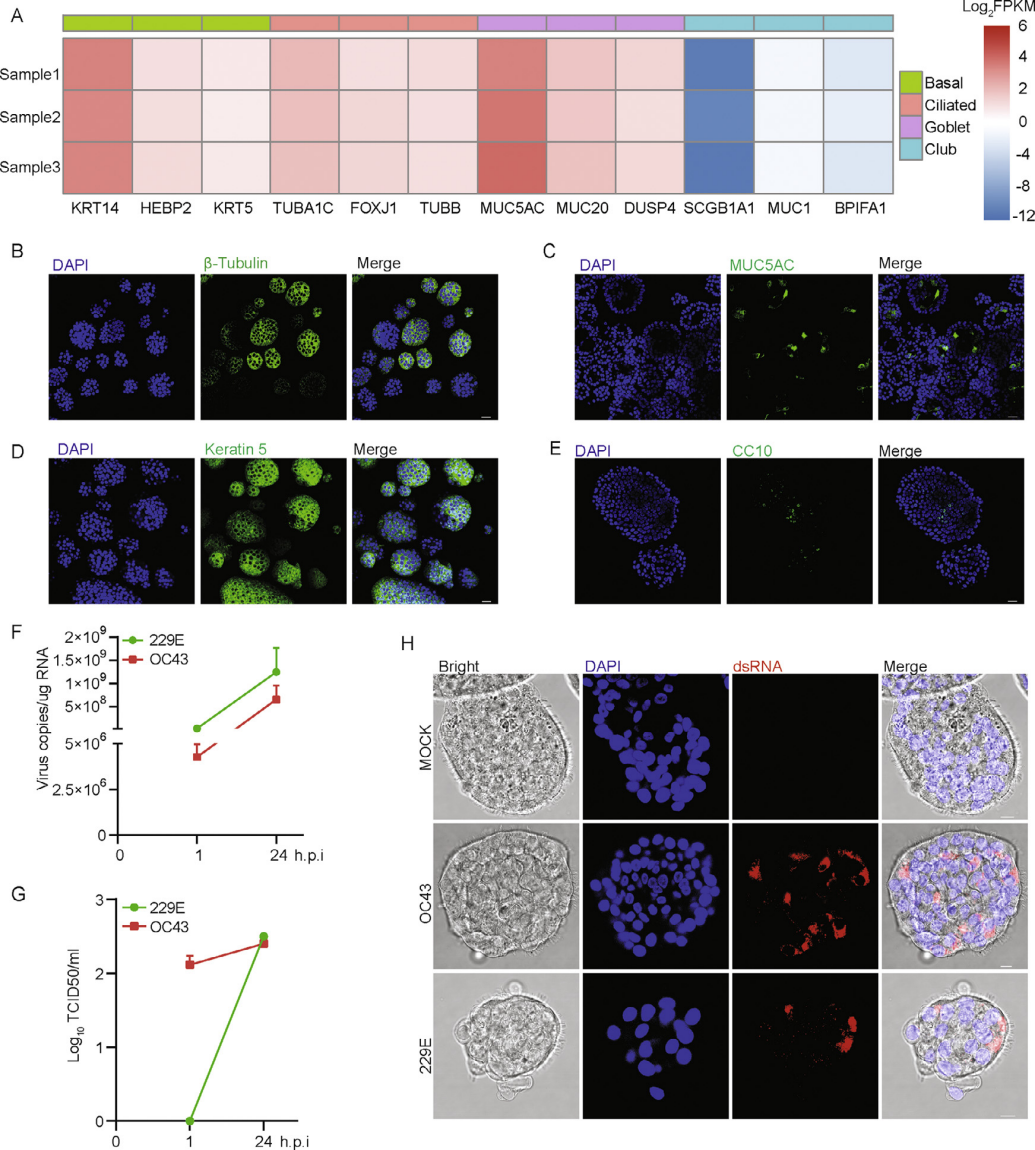
Recapitulating 229E and OC43 infections in differentiated hAOs. (A) Transcriptional levels of specific cell-type markers upon differentiation at 37 °C. (B) to (E) Characterizing cell types by immunostaining β-tubulin (ciliated cells), MUC5AC (goblet cells), Kertain 5 (basal cells) and CC10 (club cells) (Scale bar = 25 μm). (F) Dynamics of intracellular virus RNA copies in differentiated hAOs at 33 °C from 1 to 24 h post-inoculation (*n*=3). (G) Dynamics of infectious viral titers in differentiated hAOs at 33 °C from 1 to 24 h post-inoculation (*n*=3). (H) Immunofluorescence staining for viral double-stranded RNA (dsRNA) in differentiated organoids at 33 °C (Scale bar = 10 μm).

Next, differentiated hAOs were tested for the permissiveness to 229E and OC43 infections. Upon two weeks of differentiation in culture, the transcriptional level of ciliated cell markers, basal cell markers as well as goblet cell markers were dramatically upregulated, whereas club cell markers were decreased (Figure 2A). Immunofluorescence staining showed more ciliated cells (β-tubulin positive) but fewer club cells (CC10 positive) (Figure 2B to E). Interestingly, a large proportion of airway organoid cells acquired morphology with waving villus (Figure 2H). Consistently, upon inoculation of 229E and OC43 virus particles, both viral RNA levels and infectious virus titers were significantly increased within 24 h (Figure 2 F and G). Robust replication was further confirmed in ciliated organoid cells by immunostaining the viral dsRNA (Figure 2H). To further map the cell tropism of 229E and OC43, we performed co-staining using human intravenous immunoglobulin (IVIg) to detect the virus, and specific cell markers to distinguish cell types. As shown, 229E and OC43 were able to infect basal cells (Keratin 5 positive), ciliated cells (β-tubulin positive) and club cells (CC10 positive), but rarely goblet cells (MUC5AC positive) (Supplementary Figure 3). Moreover, by inoculating differentiated hAOs with NL63 at 33 °C, we demonstrated that differentiated hAOs also support NL63 virus replication and production, as showed by qPCR and immunostaining (Supplementary Figure 4).

**Figure 3:**
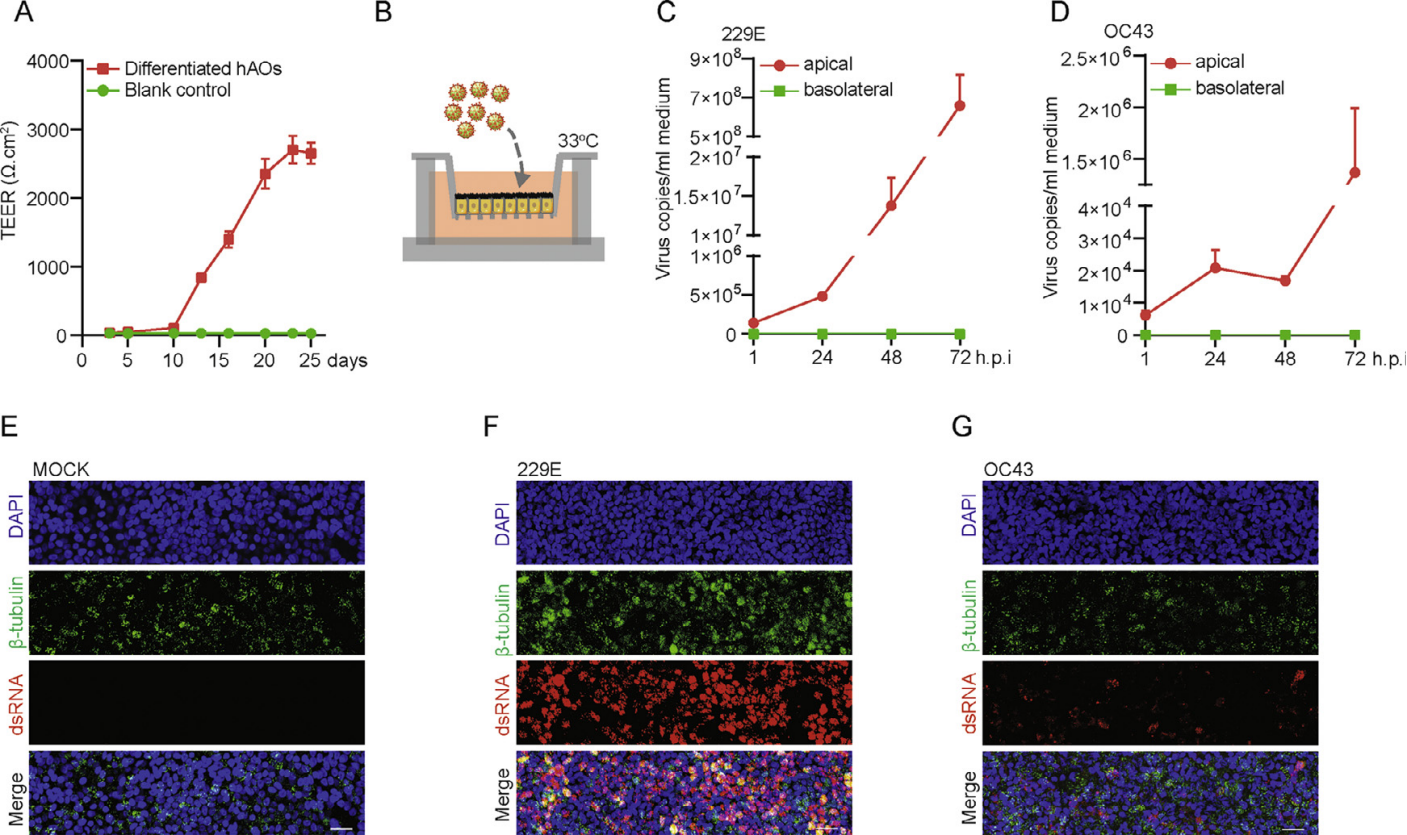
(A) TEER value of monolayers in transwell membrane tested at 3, 5, 10, 13, 16, 20, 23 and 25 days after seeding. (B) Schematic representation of virus inoculation in organoid cells in transwell system. (C) Quantification of released viruses from monolayers inoculated with 229E particles. (D) Quantification of released viruses from monolayers inoculated with OC43 particles. (E) to (G) Immunofluorescence staining for viral dsRNA and β-tubulin (ciliated cell marker) in transwell monolayers at 72 h post-inoculation (Scale bar = 25 μm).

Next, undifferentiated hAOs cultured in 3D were transformed into monolayer by seeding in transwell inserts and further induced to differentiated phenotype. The transepithelial electrical resistance (TEER) of monolayers was stabilized after 3 weeks in culture (Figure 3A). The stabilized monolayers were then inoculated with 229E and OC43 respectively from apical compartment as shown by the schematic diagram (Figure 3B). We observed predominate viral secretion into the apical but not basolateral compartment (Figure 3C and D). Confocal microscopy demonstrated the abundant presence of viral dsRNA, but the level is much higher in 229E infected cells (Figure 3E to G). Taken together, both undifferentiated and differentiated hAOs efficiently support the propagation of HCoVs 229E, OC43 and NL63.

**Figure 4:**
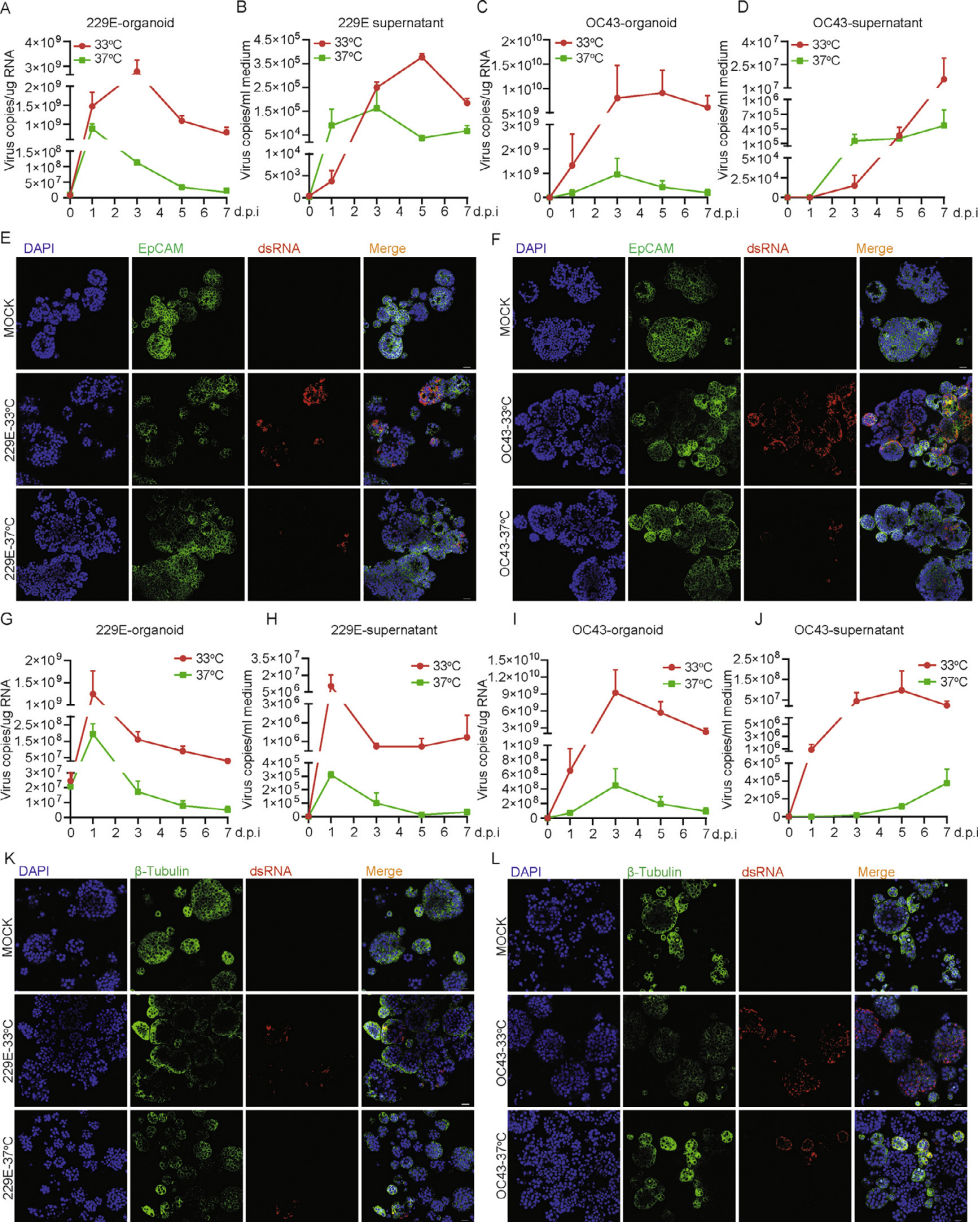
Kinetics of 229E and OC43 virus replication and production at different temperatures. (A) 229E replication kinetics in undifferentiated hAOs at 33 °C and 37 °C at 1 h, 1 day, 3 day, 5 day, 7 day post-inoculation (*n*=4). (B) The kinetics of 229E virus production (*n*=4). (C) The kinetics of OC43 viral replication (*n*=3–4). (D) The kinetics of OC43 virus production (*n*=3–4). (E) Immunofluorescence staining of 229E viral dsRNA and EpCAM (epithelial membrane marker) in undifferentiated hAOs at different temperatures at 3 days post-inoculation. (F) Immunofluorescence staining of OC43 viral dsRNA and EpCAM in undifferentiated hAOs. The kinetics of 229E viral replication (G) and production (H) in differentiated hAOs at 33 °C and 37 °C at 1 h, 1 day, 3 days, 5 days, 7 days post-inoculation (*n*=2–3). The kinetics of OC43 replication (I) and production (J) in differentiated hAOs (*n*=2–3). (K) and (L) Immunofluorescence staining of viral dsRNA and EpCAM in differentiated hAOs at 33 °C and 37 °C at 3 days post-inoculation. Scale bar = 25 μm.

To assess the impact of temperature on HCoVs propagation, the inoculated hAOs with undifferentiated phenotype were incubated at either 33 °C or 37 °C to mimic the temperature of the upper and lower respiratory tract. We found that 229E infection resulted in over 10-fold higher intracellular virus copies at 33 °C compared to 37 °C since 3 days post-inoculation. Consistently, more extracellular viruses were produced at 33 °C than that at 37 °C after 3 days accumulation in culture (Figure 4A and B). Similar results were observed when inoculating with OC43 virus particles (Figure 4C and D). Confocal microscopy revealed much more abundant numbers of dsRNA-positive cells at 33 °C compared to 37 °C (Figure 4E and F). This temperature-dependent viral propagation was further confirmed by quantifying infectious virus titer using the TCID50 assay (Supplementary Figure 5). Similarly, NL63 also replicates more efficiently at 33 °C compared to 37 °C in undifferentiated hAOs (Supplementary Figure 2A). This temperature-related disparity of permissiveness was further confirmed in differentiated hAOs (Figure 4G to L). Morphological observation during the infection period revealed massive abnormalities and disintegrations of 229E or OC43 infected organoids, especially after 5 days of infection cultured at 33 °C (Supplementary Figures 6 and 7). Propidium iodide (PI) and calcein staining marked the wide-spread of dead cells in infected organoids at both temperatures but complete disintegration of organoids mainly occurred at 33 °C (Supplementary Figure 8). Thus, we postulate that 229E, OC43 and NL63 can more efficiently replicate in upper respiratory tract with a lower temperature of 33 °C, and the infection may lead to severe cytopathogenisis.

**Figure 5:**
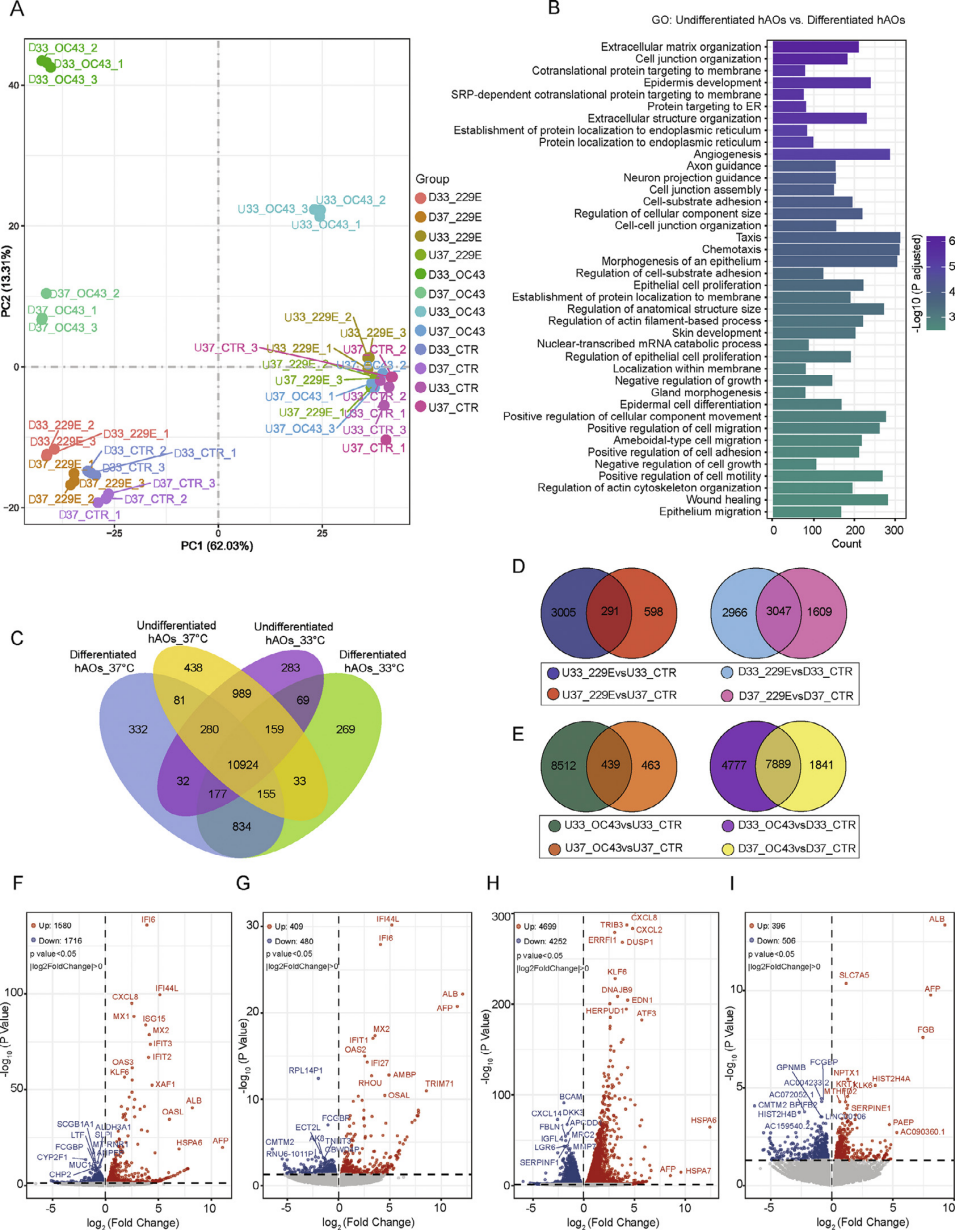
Genome-wide transcriptomic analysis in human airway organoids upon seasonal coronavirus infection. (A) Principal component analysis (PCA) of different organoid samples (U: Undifferentiated hAOs; D: Differentiated hAOs). (B) Top 40 significantly enriched pathways by gene ontology (GO) analysis of hAOs cultured at 37 °C. (C to E) Venn diagram of overlapped differentially expressed genes (C) in differentiated and undifferentiated airway organoids cultured at 33 °C and 37 °C, (D) 229E virus infection in undifferentiated hAOs or differentiated hAOs cultured at 33 °C and 37 °C, (E) OC43 virus infection in undifferentiated hAOs or differentiated hAOs cultured at 33 °C and 37 °C. (F to I) Differential gene expression analysis by coronavirus infections in undifferentiated hAOs, (F) 229E infection at 33 °C, (G) 229E infection at 37 °C, (H) OC43 infection at 33 °C, (I) OC43 infection at 37 °C.

To better understand host responses upon coronavirus infections, genome-wide transcriptomic analysis was performed at various conditions. Principal component analysis (PCA) clearly separated undifferentiated from differentiated hAOs (Figure 5A). Gene oncology enrichment analysis of differentially expressed (DE) genes between undifferentiated and differentiated hAOs revealed the major regulated pathways belong to cell development and morphogenesis, indicating the distinct cellular characteristics between these two types of hAOs (Figure 5B). Comparison of the DE genes among undifferentiated and differentiated hAOs that were cultured at two temperature conditions showed that hundreds of genes were exclusively expressed in each group, but over 10,000 genes were shared among the four groups (Figure 5C). Similarly, thousands of DE genes upon 229E and OC43 infection were identified specific to the temperature condition regardless of the differentiation status of hAOs (Figure 5D and E). Upon 229E and OC43 infections, volcano plot revealed that the amount of significantly up- or down-regulated genes in undifferentiated hAOs are 3-10 fold more when culturing at 33 °C in comparison to culturing at 37 °C (Figure 5F to I). The most highly upregulated genes in response to 229E infection, such as IFI6, MX1, ISG15 and OAS3, were interferon-stimulated genes (ISGs), whereas OC43 elicited few ISGs. Consistently, in differentiated hAOs, 229E infection but not OC43 infection upregulated a large amount of ISGs (Supplementary Figure 9). GO analysis indicated 229E infection in hAOs triggered more robust antiviral responses such as type I interferon signaling pathway when culturing at 33°C compared to culturing at 37 °C (Supplementary Figure 10). Differently, OC43 infection elicited multiple apoptotic and catabolic related pathways. (Supplementary Figure 11).

**Figure 6:**
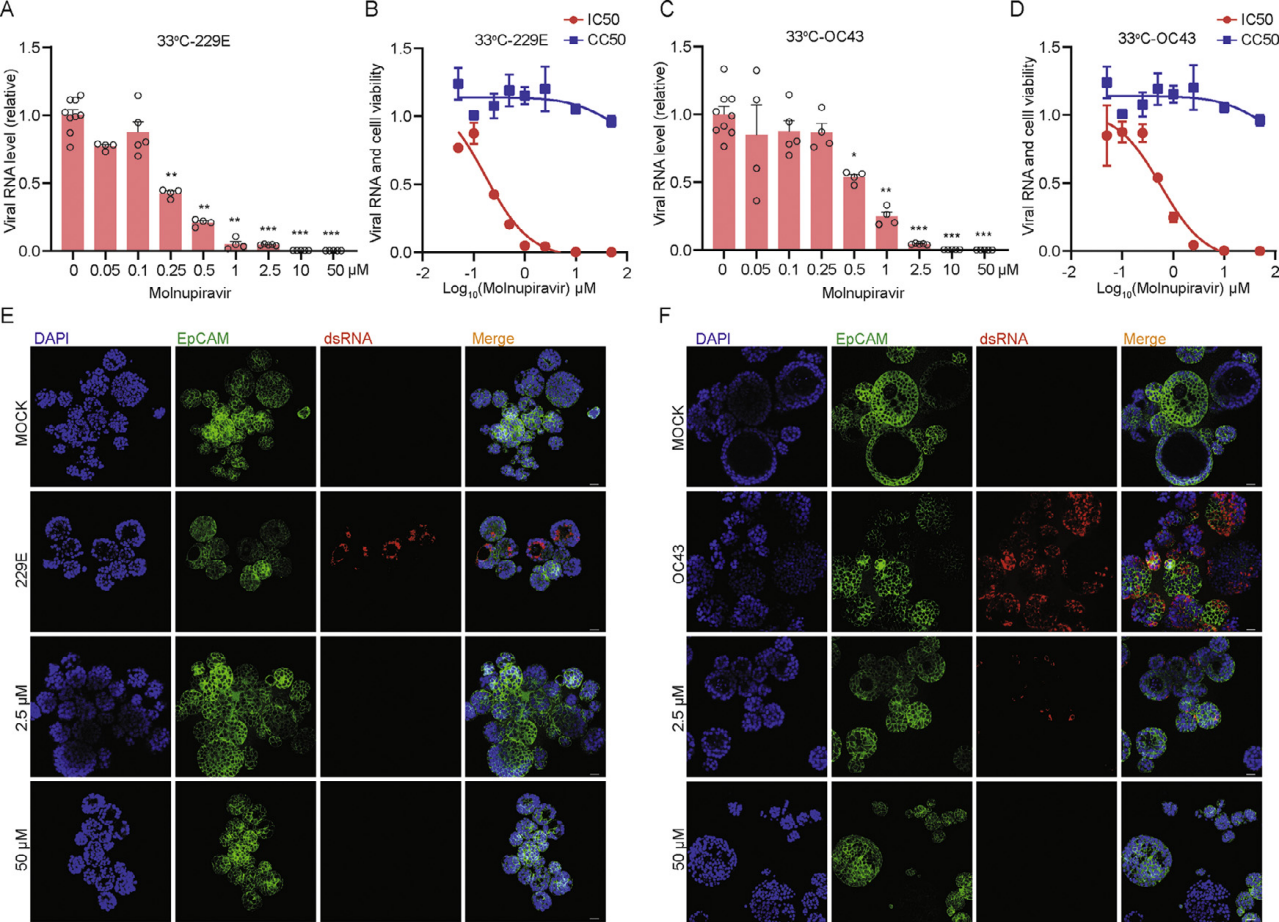
Antiviral activity of molnupiravir against 229E and OC43 in hAOs cultured at 33 °C. (A) The inhibitory effect of molnupiravir on 229E replication in hAOs cultured at 33 °C (*n* = 4–10). (B) IC50 and CC50 of molnupiravir in hAOs infected with 229E and cultured at 33 °C (*n* = 6–8). (C) The inhibitory effect of molnupiravir on OC43 replication in hAOs cultured at 33 °C (*n* = 4–9). (D) IC50 and CC50 of molnupiravir in hAOs infected with OC43 and cultured at 33 °C (*n* = 6–8). (E) and (F) The inhibitory effect of low concentration (2.5 μM) and high concentration (50 μM) of molnupiravir by immunostaining dsRNA in hAOs cultured at 33 °C. Scale bar = 25 μm. *P < 0.05; **P < 0.01; ***P < 0.001.

**Figure 7:**
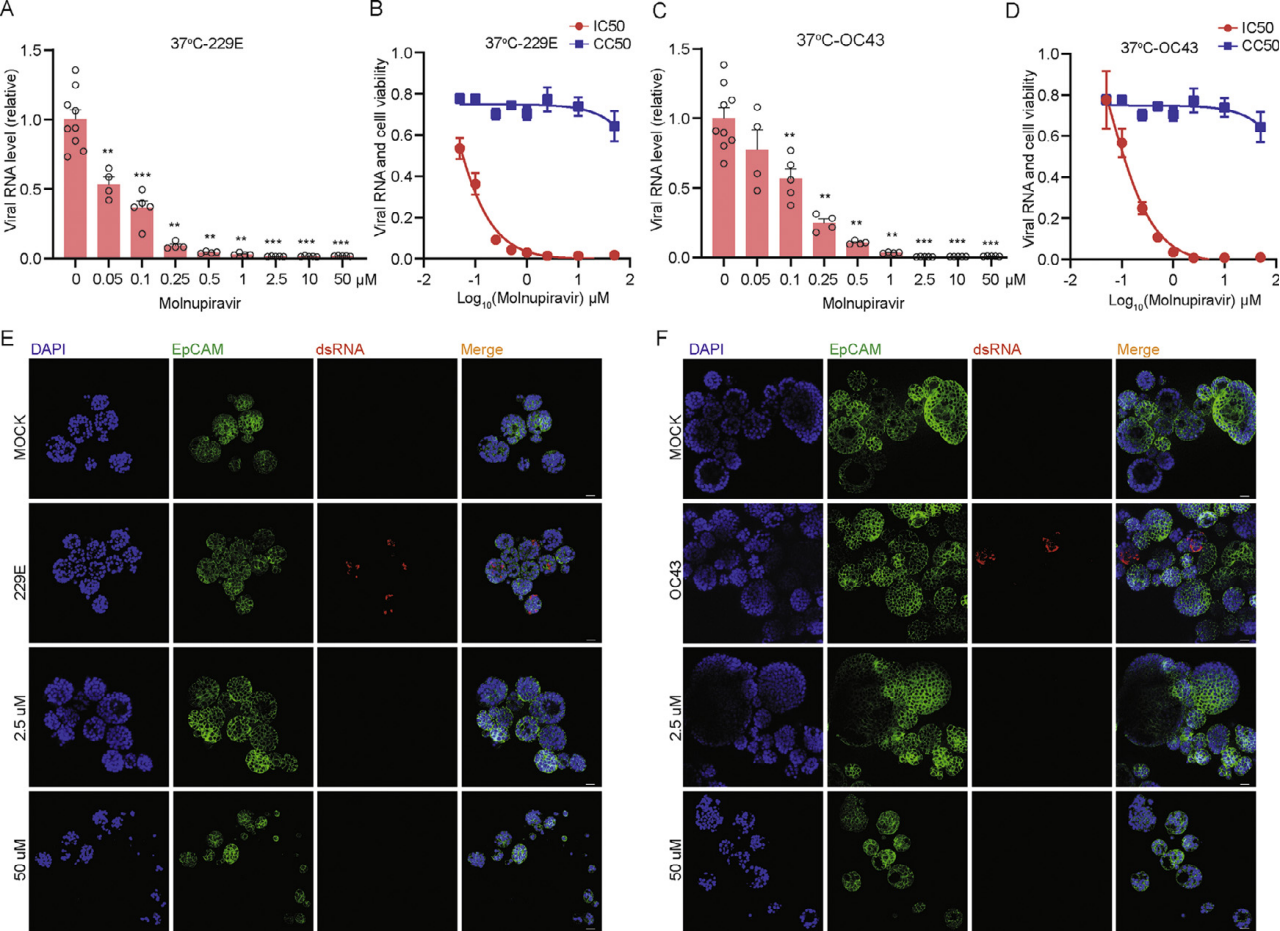
Antiviral activity of molnupiravir against 229E and OC43 in hAOs cultured at 37 °C. (A) The inhibitory effect of molnupiravir against 229E in hAOs cultured at 37 °C (*n*= 4–9). (B) IC50 and CC50 of molnupiravir in hAOs infected with 229E and cultured at 37 °C (*n* = 6–8). (C) The inhibitory effect of molnupiravir against OC43 in hAOs cultured at 37 °C (*n* = 4–9). (D) IC50 and CC50 of molnupiravir in hAOs infected with OC43 and cultured at 37 °C (*n* = 6–8). (E) and (F) The inhibitory effect of low concentration (2.5 μM) and high concentration (50 μM) of molnupiravir by immunostaining dsRNA in hAOs cultured at 37 °C. Scale bar = 25 μm. *P < 0.05; **P < 0.01; ***P < 0.001.

Since no approved treatment for seasonal HCoVs is available, we tested monupiravir and remdesivir, two antiviral agents approved for treating SARS-CoV-2 infection, in hAOs infected with 229E, OC43 and NL63. After treatment with serial dilutions of molnupiravir for 48 h at 33 °C, molnupiravir dose-dependently inhibited 229E replication in undifferentiated hAOs. A low concentration (0.25 μM) has already resulted in over 50% inhibition at viral RNA level, 1 μM concentration inhibited viral RNA by 95.0 ± 2.0% (mean ± SEM, *n* = 4, *p*= 0.0028), and complete inhibition was achieved by higher concentrations (Figure 6A). Importantly, the wide window between half maximal inhibitory concentration (IC50; 0.1712 μM) and half maximal cytotoxic concentration (CC50; noticeable cytotoxicity was absent with the tested concentrations) highlights the promising of molnupiravir for treating HCoVs infected patients (Figure 6B). Such potent antiviral activity was also observed against OC43 infection with IC50 of 0.57 μM at 33 °C (Figure 6C and D). The inhibition effect on both viruses was further confirmed by immunofluorescence staining viral dsRNA (Figure 6E and F). Next, we tested molnupiravir at 37 °C and confirmed the potent antiviral activity against both 229E and OC43 (Figure 7A to F). Molnupiravir also dose-dependently inhibited NL63 virus replication, but to a lesser extent (Supplementary Figure 12A and B). Finally, treatment of remdesivir also dose-dependently inhibited the replication of all three seasonal coronaviruses (Supplementary Figure 12C to E). These results collectively support the exploration of repurposing molnupiravir and remdesivir for treating seasonal HCoVs infections.

## Discussion

Robust *in vitro* systems that can efficiently support viral infections and resemble *in vivo* characteristics are essentially required for understanding the pathogenesis and developing treatment. Immortalized cell lines are widely used for modeling viral infections but have many intrinsic limitations. The advent of organoid technique provides unique platforms to authentically recapitulate virus-host interactions.^15^ Airway organoids can physiologically resemble the respiratory tract, which can self-organize *in vitro* to form ‘mini-airways’, with outer layers lined by basal cells and inner lumen constructed by cilia, secretory cells and club cells.^16^ These features are ideal in reflecting the subtle changes of *in vivo* microenvironment during respiratory virus replication and propagation.^13^^,^^17^ Extending the previous research of using hAOs for SARS-CoV-2 infection,^13^^,^^14^ this study established successful infections of seasonal HCoVs 229E, OC43 and NL63 in hAOs with both undifferentiated and differentiated phenotypes cultured in 2D or 3D. In 2D cultured transwell system, we observed predominantly apical release of the viruses, mimicking viral dissemination from respiratory tract in infected patients. So far, there are four species of seasonal HCoVs (NL63, 229E, OC43 and HKU1) identified, but the research into these viruses is limited and robust experimental models are largely lacking. Viruses entry host cells by binding to specific receptors. 229E is known to utilize aminopeptidase (ANPEP) as a receptor to enter and access host cells.^18^ We found that ANPEP expression is thousand fold higher in undifferentiated compared to differentiated hAOs, which may partially explain why 229E propagates more efficiently in undifferentiated hAOs (Supplementary Figure 13). NL63 is the only member that utilizes angiotensin converting enzyme 2 (ACE2) as its receptor, similar to SARS-CoV-1 and SARS-CoV-2.^19^ ACE2 expression was upregulated by over 20-fold in hAOs after differentiation (Supplementary Figure 13). The HKU1 species is very difficult to be cultured *in vitro* and future isolation of more permissive strains would allow the possibility of establishing infection in organoids.

Respiratory viruses, such as influenza viruses, coronaviruses and rhinoviruses, have been shown to be more permissive to cooler temperature.^7^, ^8^, ^9^ Although the underlying mechanisms are largely unknown, host immunity has been suggested as one of the key players. Higher body temperatures have been shown to boost host immune response during infections, which in turn constrain viral replication.^20^ In this study, we found seasonal HCoVs replicate more efficiently at 33 °C (resembling the temperature at upper airway) compared to 37 °C (the temperature at lower airway). This temperature-dependent pattern of viral growth is in line with the clinical manifestations that most seasonal HCoVs infections cause upper respiratory illness.^21^ This partiality to cooler temperature might promote the transmissibility of coronaviruses, which has been observed in SARS-CoV-2.^7^ Although less permissive at 37 °C, moderate levels of viral replication and cytopathogenisis especially by OC43 infection, were observed in organoids upon infection at this temperature.

The underlying mechanisms orchestrating temperature-dependent permissiveness of respiratory viral infections remain largely elusive. Rhinovirus, a major cause of common cold, was found to be more efficiently replicates at 33 °C compared to 37 °C, attributing to diminished antiviral immune responses at cooler temperature.^9^ In contrast, we observed a more robust host response provoked by 229E infection at 33 °C compared to 37 °C, in particular type I interferon (IFN) response with most of the top upregulated genes as ISGs. IFNs are the first-line defense against viral infections. However, IFN response was not prominent in hAOs upon OC43 infection. This is in line with a previous study showing that OC43 can effectively counteract antiviral IFN response.^22^ Nevertheless, our transcriptomic analysis revealed that OC43 infection enriched multiple apoptotic-related pathways in hAOs, another mechanism of antiviral defense. This is also consistent with the severe cytopathogenisis upon OC43 infection as we observed.

Molnupiravir has been shown to potently inhibit SARS-CoV-2 through introducing copying errors during viral RNA replication.^23^ Considering favorable safety and efficacy profiles in clinical trials, it has been expeditiously approved for treating COVID-19 as the first oral antiviral agent.^24^ Our previous study has demonstrated the pan-coronavirus antiviral activity of molnupiravir in molecular docking and in various cell line models.^25^ Here, we further confirmed the potent antiviral activity of molnupiravir against 229E, OC43 and NL63 in hAOs models at 33 °C and 37 °C. Remdesivir was the first approved antiviral drug for treating COVID-19, but it has to be administered intravenously. It binds to viral RNA-dependent RNA polymerase and inhibits viral replication by terminating RNA transcription prematurely.^26^ We demonstrated the inhibition of seasonal coronaviruses in hAOs by remdesivir treatment. However, remdesivir appears to be more effective in inhibiting NL63, whereas molnupiravir is more potent in inhibiting 229E and OC43 replication. Given the lacking of specific treatment, we emphasize the great potential of repurposing molnupiravir and remdesivir for treating severe seasonal HCoVs infections, although further validation *in vivo* is required.

In this study, we highlight human airway organoids as innovative models for seasonal coronavirus infections, but there are also other *in vitro* and *in vivo* models available and we do not object to the use of conventional cell lines.^27^^,^^28^ For example, immortalized cell lines have been essential for initial isolation and propagation of coronaviruses from patient samples.^29^ We have recently demonstrated that organoids model is feasible for small- to medium-scale drug screening,^27^ but large-scale drug screening remains rely on cell line models.^30^ Compared to *in vitro* models, animals are often better in recapitulating the complexity of viral infections and clinical presentations as seen in patients.^31^ However, there are major ethical concerns of using experimental animals. We advocate further improvement of human organoid-based models, for example adding immune and stromal cell compartments into organoids, for better recapitulating the critical features of the disease and facilitating drug development.^32^

In summary, we have successfully established airway organoids-based models for seasonal HCoVs 229E, OC43 and NL63 infections. We have demonstrated temperature-dependent regulation of viral replication, and the potent antiviral activity of molnupiravir and remdesivir in hAOs. These innovative models shall help to advance the research and therapeutic development against seasonal HCoVs infections.

## Contributors

Conceptualization, P.L., B.L.H, and Q.P.; Methodology, P.L., Y.W., and M.L.; Resources, M.M.L., I.C., and R.J.R; Formal analysis, P.L., and Y.W.; Writing-Original Draft, P.L. and Q.P.; Funding acquisition, Q.P. and R.J.R.; Writing-Review & Editing, M.M.L., I.C., A.C.V., R.J.R., M.P.P., B.L.H., and Q.P.; P.L. and Q.P. have accessed and verified the data. All authors read and approved the final version of the manuscript.

## Data sharing statement

All the data supporting the findings of this study are available within the article and its Supplemental Information files.

## Declaration of interests

No conflict of interest.

## Appendix Supplementary materials

Image, application 1

## References

[1] Sariol A, Perlman S. (2020). Lessons for COVID-19 immunity from other coronavirus infections. Immunity.

[2] Ma Z, Li P, Ji Y, Ikram A, Pan Q. (2020). Cross-reactivity towards SARS-CoV-2: the potential role of low-pathogenic human coronaviruses. Lancet Microbe.

[3] Li P, Liu J, Ma Z, Bramer WM, Peppelenbosch MP, Pan Q. (2020). Estimating global epidemiology of low-pathogenic human coronaviruses in relation to the COVID-19 context. J Infect Dis.

[4] Veiga A, Martins LG, Riediger I, Mazetto A, Debur MDC, Gregianini TS. (2021). More than just a common cold: endemic coronaviruses OC43, HKU1, NL63, and 229E associated with severe acute respiratory infection and fatality cases among healthy adults. J Med Virol.

[5] Man WH, de Steenhuijsen Piters WA, Bogaert D. (2017). The microbiota of the respiratory tract: gatekeeper to respiratory health. Nat Rev Microbiol.

[6] McFadden ER, Pichurko BM, Bowman HF (1985). Thermal mapping of the airways in humans. J Appl Physiol (1985).

[7] V'Kovski P, Gultom M, Kelly JN (2021). Disparate temperature-dependent virus-host dynamics for SARS-CoV-2 and SARS-CoV in the human respiratory epithelium. PLoS Biol.

[8] Holwerda M, Kelly J, Laloli L (2019). Determining the replication kinetics and cellular tropism of influenza D virus on primary well-differentiated human airway epithelial cells. Viruses.

[9] Foxman EF, Storer JA, Fitzgerald ME (2015). Temperature-dependent innate defense against the common cold virus limits viral replication at warm temperature in mouse airway cells. Proc Natl Acad Sci USA.

[10] Dutta D, Heo I, Clevers H. (2017). Disease modeling in stem cell-derived 3D organoid systems. Trends Mol Med.

[11] Sachs N, Papaspyropoulos A, Zomer-van Ommen DD (2019). Long-term expanding human airway organoids for disease modeling. EMBO J.

[12] Zhou J, Li C, Sachs N (2018). Differentiated human airway organoids to assess infectivity of emerging influenza virus. Proc Natl Acad Sci USA.

[13] Salahudeen AA, Choi SS, Rustagi A (2020). Progenitor identification and SARS-CoV-2 infection in human distal lung organoids. Nature.

[14] Lamers MM, Beumer J, van der Vaart J (2020). SARS-CoV-2 productively infects human gut enterocytes. Science.

[15] Dutta D, Clevers H. (2017). Organoid culture systems to study host-pathogen interactions. Curr Opin Immunol.

[16] Hui KPY, Ching RHH, Chan SKH (2018). Tropism, replication competence, and innate immune responses of influenza virus: an analysis of human airway organoids and ex-vivo bronchus cultures. Lancet Respir Med.

[17] de Oliveira M, De Sibio MT, Costa FAS, Sakalem ME. (2021). Airway and alveoli organoids as valuable research tools in COVID-19. ACS Biomater Sci Eng.

[18] Li Z, Tomlinson AC, Wong AH (2019). The human coronavirus HCoV-229E S-protein structure and receptor binding. Elife.

[19] Hofmann H, Pyrc K, van der Hoek L, Geier M, Berkhout B, Pohlmann S. (2005). Human coronavirus NL63 employs the severe acute respiratory syndrome coronavirus receptor for cellular entry. Proc Natl Acad Sci USA.

[20] Evans SS, Repasky EA, Fisher DT. (2015). Fever and the thermal regulation of immunity: the immune system feels the heat. Nat Rev Immunol.

[21] Li P, Ikram A, Peppelenbosch MP, Ma Z, Pan Q. (2021). Systematically mapping clinical features of infections with classical endemic human coronaviruses. Clin Infect Dis.

[22] Zhao X, Guo F, Liu F (2014). Interferon induction of IFITM proteins promotes infection by human coronavirus OC43. Proc Natl Acad Sci USA.

[23] Wahl A, Gralinski LE, Johnson CE (2021). SARS-CoV-2 infection is effectively treated and prevented by EIDD-2801. Nature.

[24] Fischer W, Eron JJ, Holman W, et al. A phase 2a clinical trial of molnupiravir in patients with COVID-19 shows accelerated SARS-CoV-2 RNA clearance and elimination of infectious virus, *Sci Transl Med*. 2022;14(628):eabl7430. 10.1126/scitranslmed.abl7430 PMC10763622 34941423

[25] Wang Y, Li P, Solanki K (2021). Viral polymerase binding and broad-spectrum antiviral activity of molnupiravir against human seasonal coronaviruses. Virology.

[26] Wang M, Cao R, Zhang L (2020). Remdesivir and chloroquine effectively inhibit the recently emerged novel coronavirus (2019-nCoV) in vitro. Cell Res.

[27] Li P, Li Y, Wang Y (2022). Recapitulating hepatitis E virus-host interactions and facilitating antiviral drug discovery in human liver-derived organoids. Sci Adv.

[28] Yin Y, Bijvelds M, Dang W (2015). Modeling rotavirus infection and antiviral therapy using primary intestinal organoids. Antiviral Res.

[29] Zhou P, Yang XL, Wang XG (2020). A pneumonia outbreak associated with a new coronavirus of probable bat origin. Nature.

[30] Riva L, Yuan S, Yin X (2020). Discovery of SARS-CoV-2 antiviral drugs through large-scale compound repurposing. Nature.

[31] Munoz-Fontela C, Dowling WE, Funnell SGP (2020). Animal models for COVID-19. Nature.

[32] Wang L, Li M, Yu B (2022). Recapitulating lipid accumulation and related metabolic dysregulation in human liver-derived organoids. J Mol Med (Berl).

