# Supplementary material for: Recapitulating infection, thermal sensitivity and antiviral treatment of seasonal coronaviruses in human airway organoids
Source: eBioMedicine. 2022 Jun 29;81:104132. doi: 10.1016/j.ebiom.2022.104132 (PMC9240613; doi:10.1016/j.ebiom.2022.104132)
Supplement: Supplementary file 1 [file mmc1.docx]

**Recapitulating infection, thermal sensitivity and antiviral treatment of seasonal coronaviruses in human airway organoids**

*P. Li et al.*

Figure S1


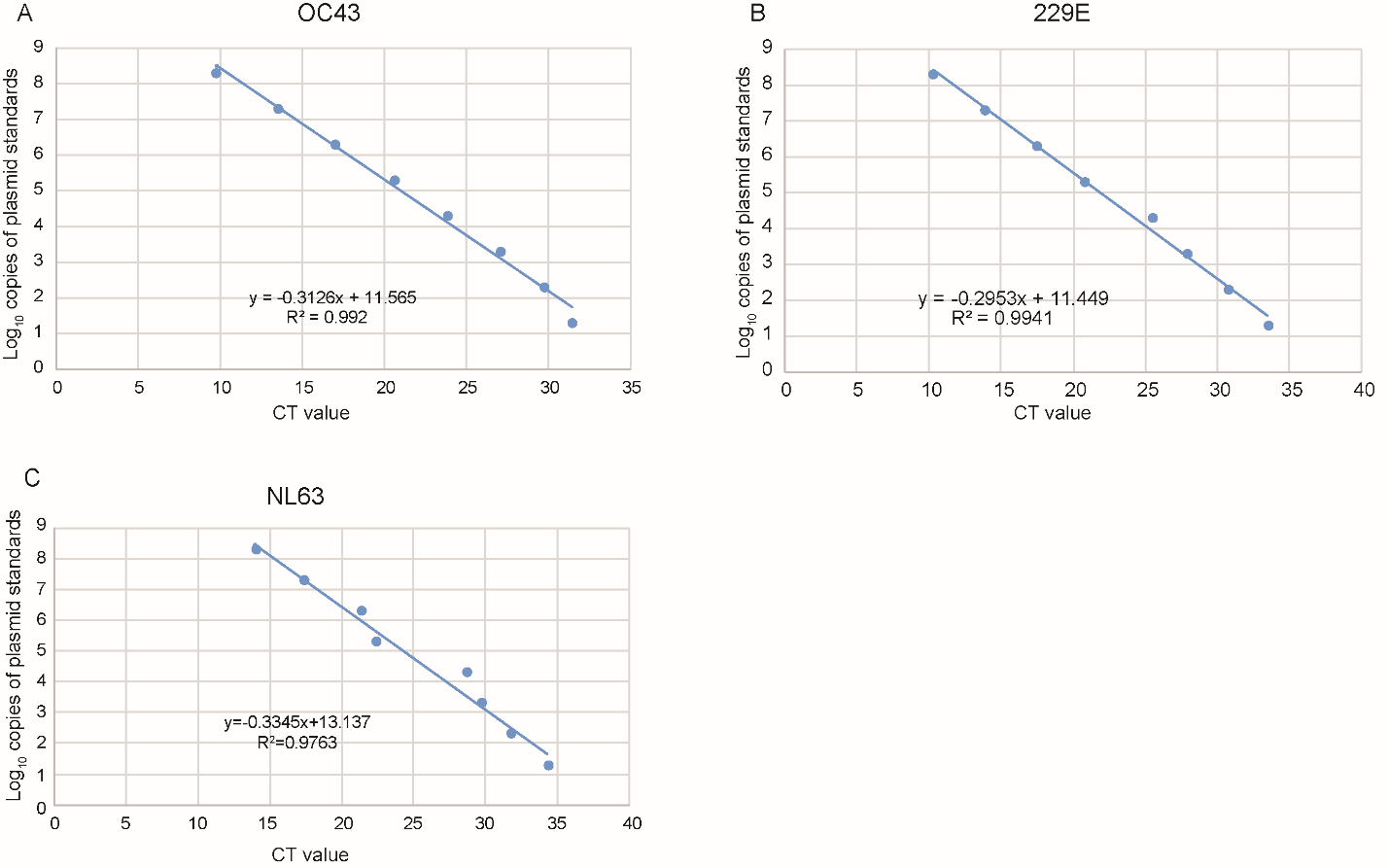


**Figure S1.** Standard curve for quantification of OC43, 229E and NL63 genome copy numbers. Plasmids containing corresponding HCoV partial genome were diluted from 10^-1^ to 10^-9^, and amplified and quantified by qRT-PCR.

Figure S2


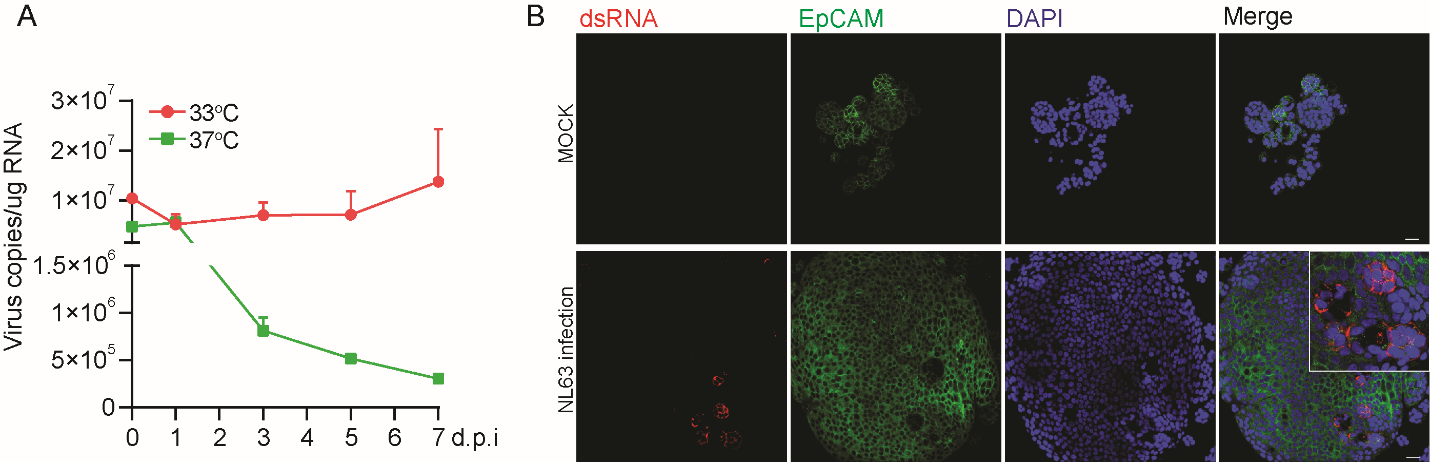


Figure S2. Recapitulating NL63 infections in undifferentiated hAOs. (A) Dynamics of intracellular NL63 virus RNA copies in undifferentiated hAOs at 33ºC and 37ºC at 1 hour, 1 day, 3 day, 5 day, 7 day post-inoculation (n=3). (B) Immunofluorescence staining of NL63 viral dsRNA and EpCAM (epithelial membrane marker) in undifferentiated hAOs. Scale bar = 25 μm

Figure S3


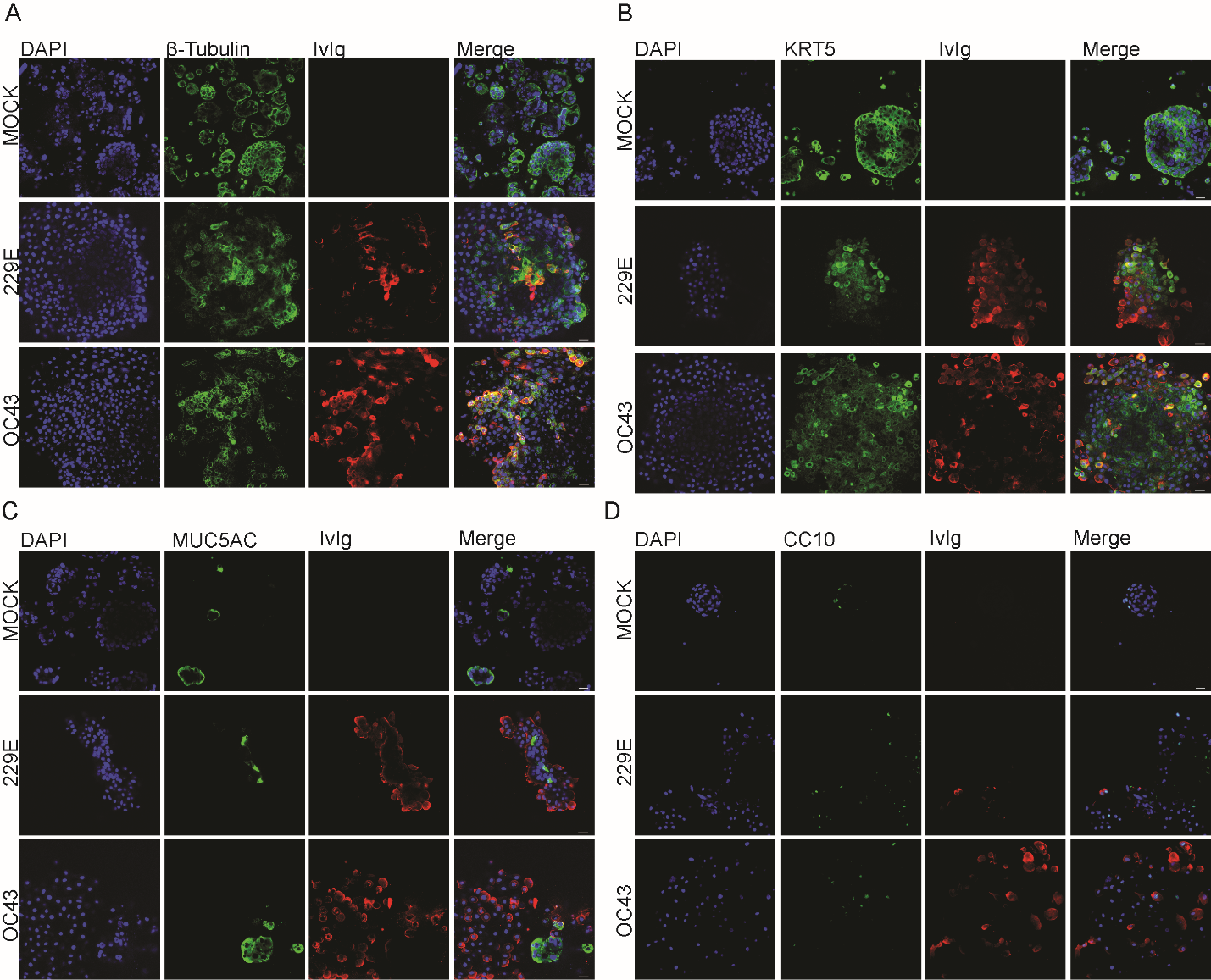


Figure S3. Characterizing the cell tropism of 229E and OC43 in differentiated hAOs. (A) to (D) Co-staining IVIg (for staining virus) and β-tubulin (ciliated cells), KRT5 (basal cells), MUC5AC (goblet cells) and CC10 (club cells). Scale bar = 25 μm

Figure S4


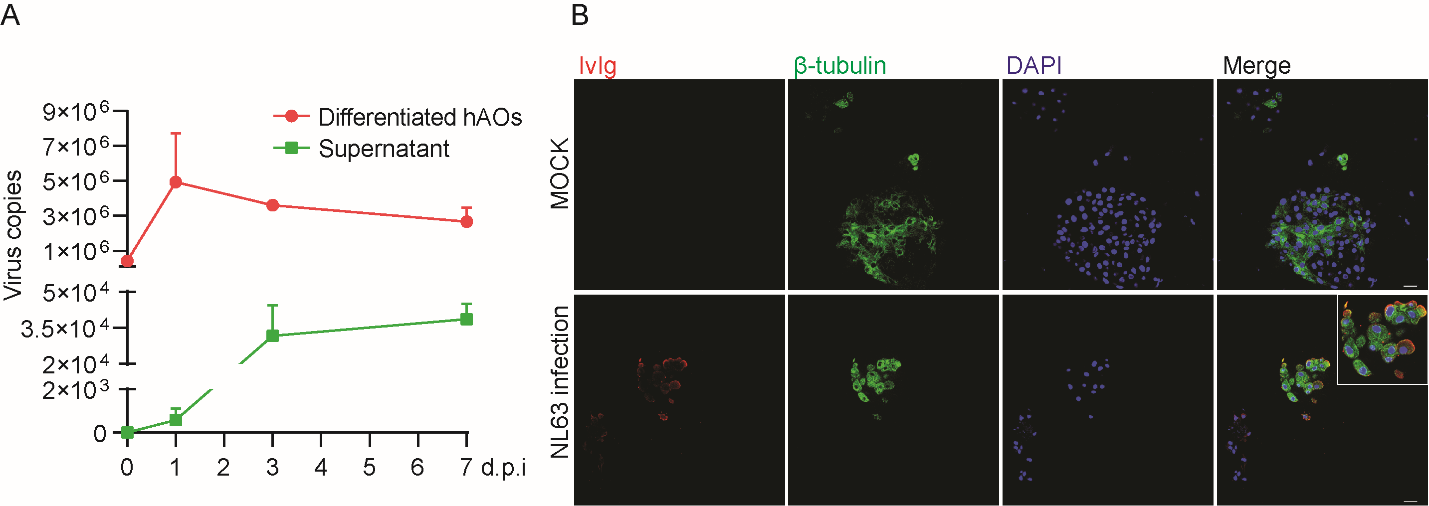


Figure S4. Recapitulating NL63 infections in differentiated hAOs. (A) Kinetics of NL63 virus replication and production in differentiated hAOs at 33ºC at 1 hour, 1 day, 3 day, 7 day post-inoculation. (B) Immunofluorescence staining of β-tubulin (ciliated cells) and IVIg (for staining virus). Scale bar = 25 μm

Figure S5


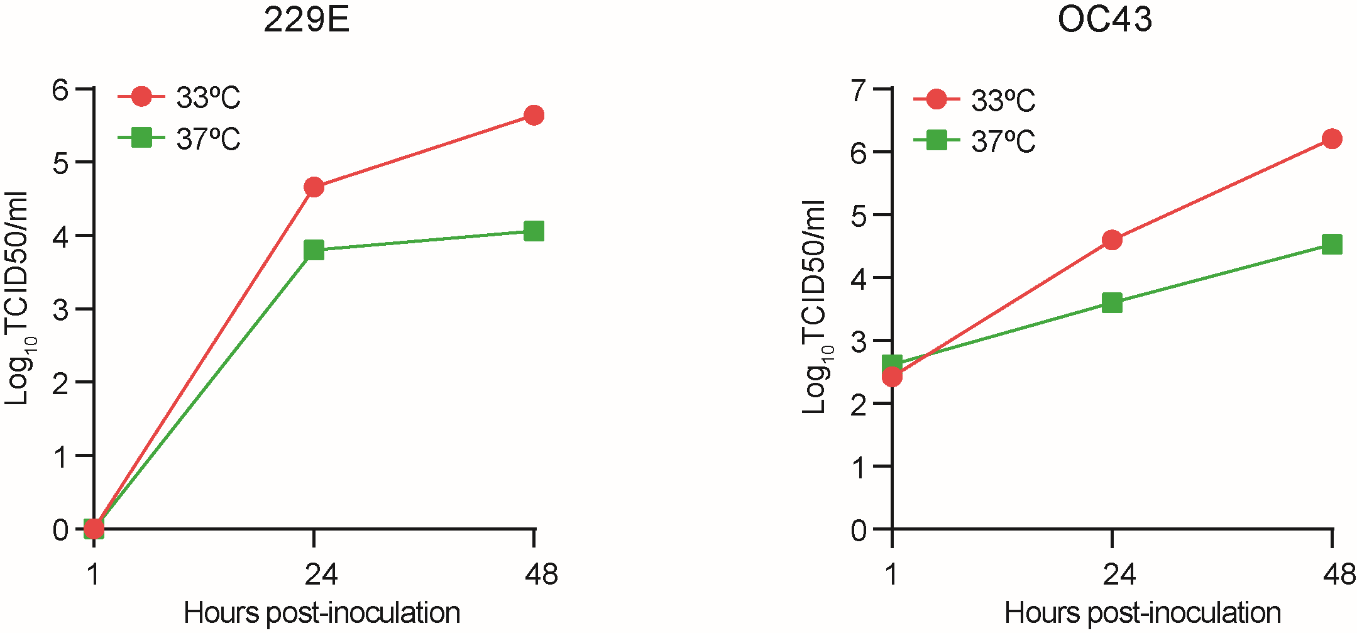


Figure S5. Dynamics of infectious viral titers in hAOs at 33ºC and 37ºC from 1 to 48 hours post-inoculation (n=3).

Figure S6


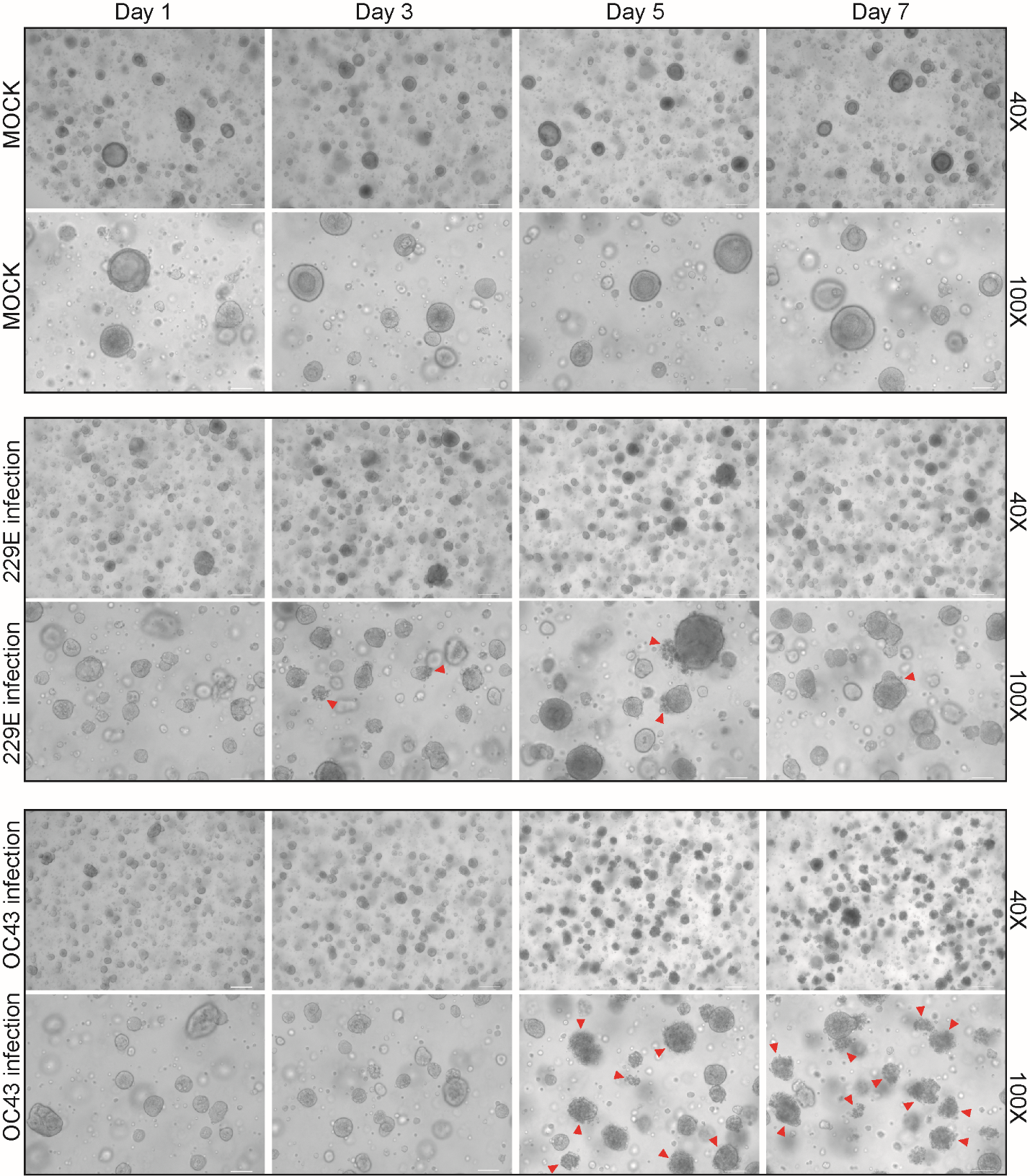


Figure S6. Morphology of undifferentiated hAOs after inoculation with 229E or OC43 cultured at 33^o^C from day 1 to day 7.

Figure S7


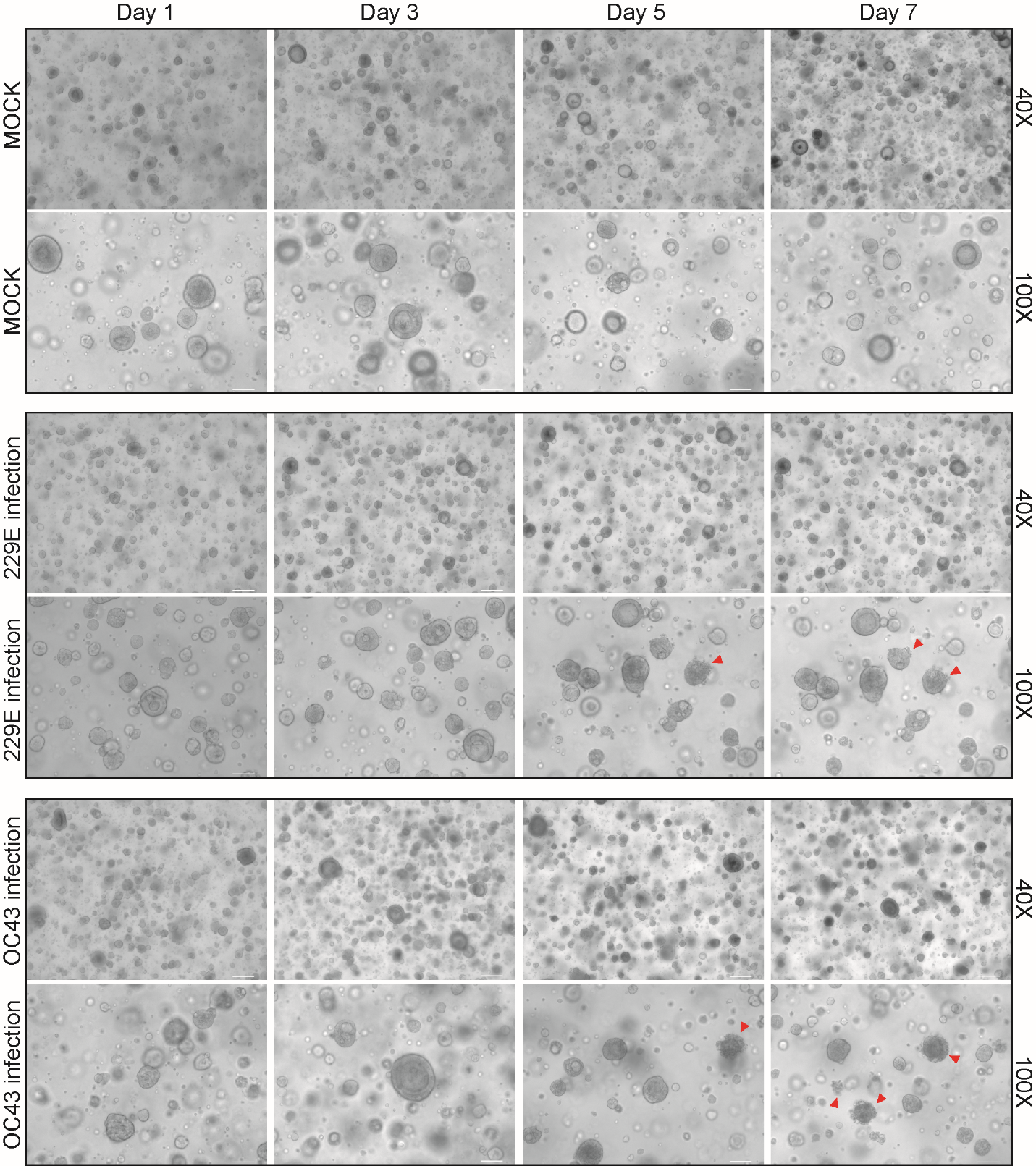


Figure S7. Morphology of undifferentiated hAOs after inoculation with 229E or OC43 cultured at 37^o^C from day 1 to day 7.

Figure S8


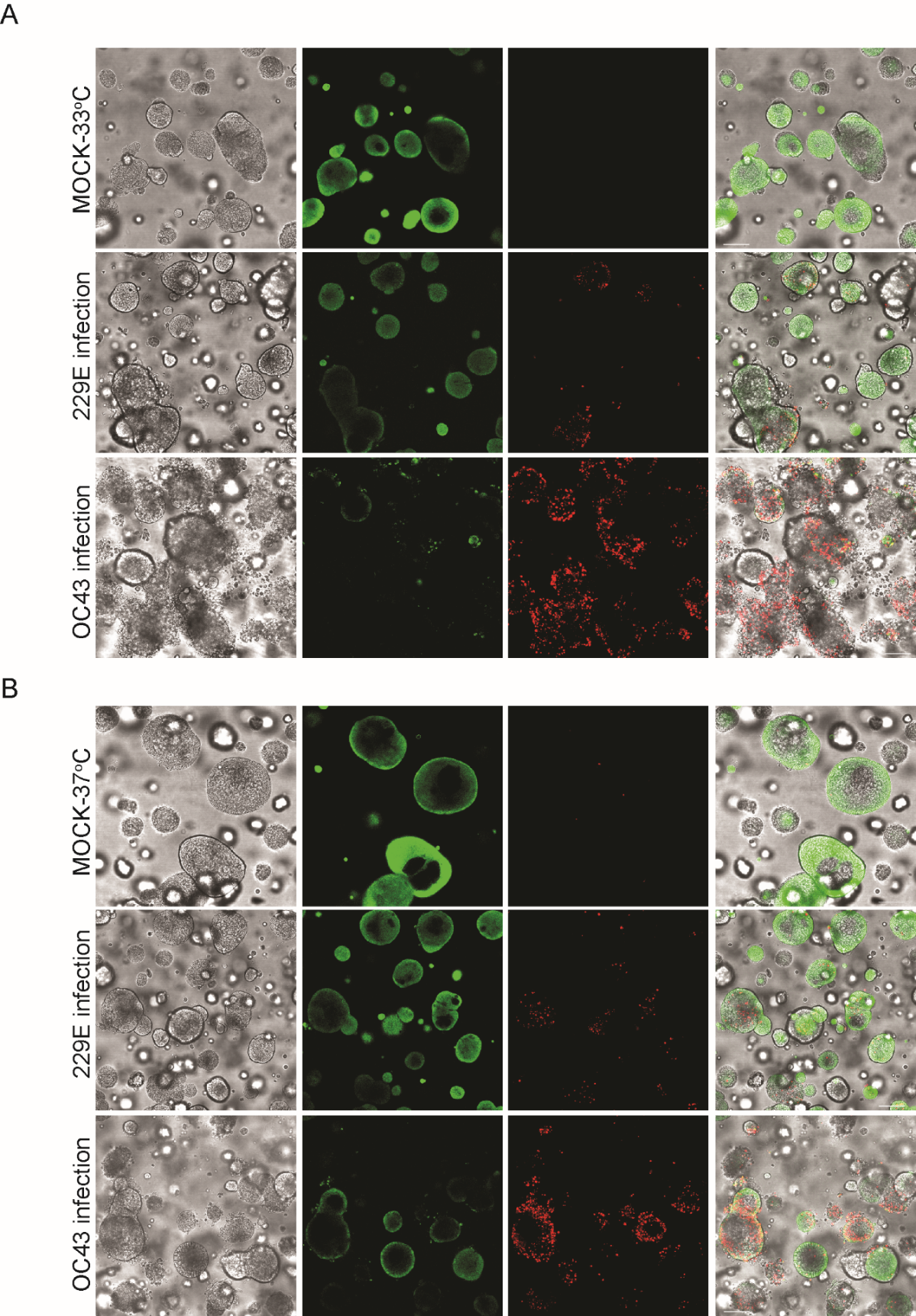


Figure S8. Fluorescence staining of dead cells (PI; red), live cells (Calcein; green) and bright field at 7 days after inoculation. (A) HAOs were cultured at 33^o^C. (B) HAOs were cultured at 37^o^C.

Figure S9


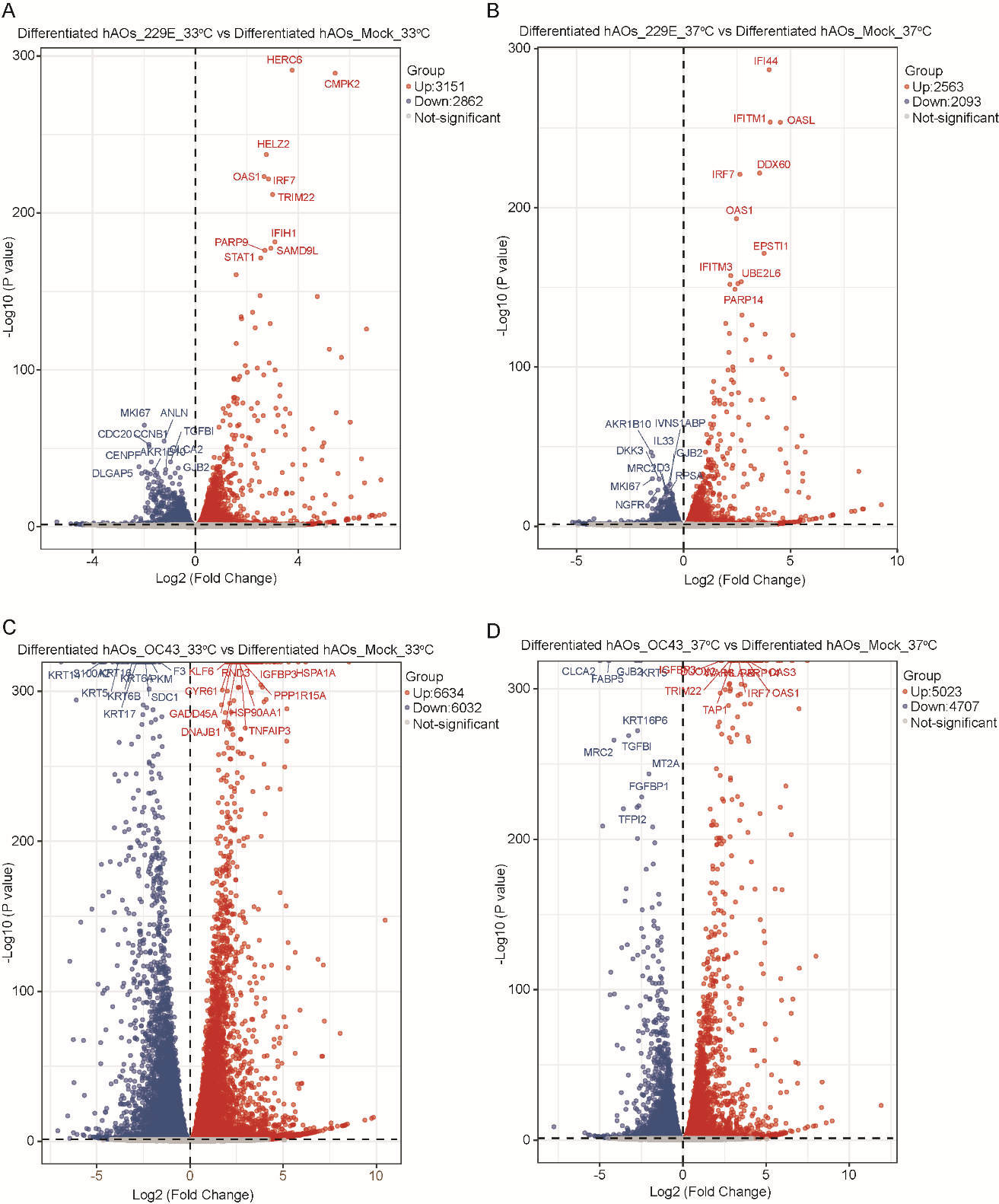


Figure S9. Differential gene expression analysis by 229E and OC43 infections in differentiated hAOs cultured at 33^o^C and 37^o^C. (A) Differential gene expression upon 229E infection at 33^o^C. (B) Differential gene expression upon 229E infection at 37^o^C. (C) Differential gene expression upon OC43 infection at 33^o^C. (D) Differential gene expression upon OC43 infection at 37^o^C.

Figure S10


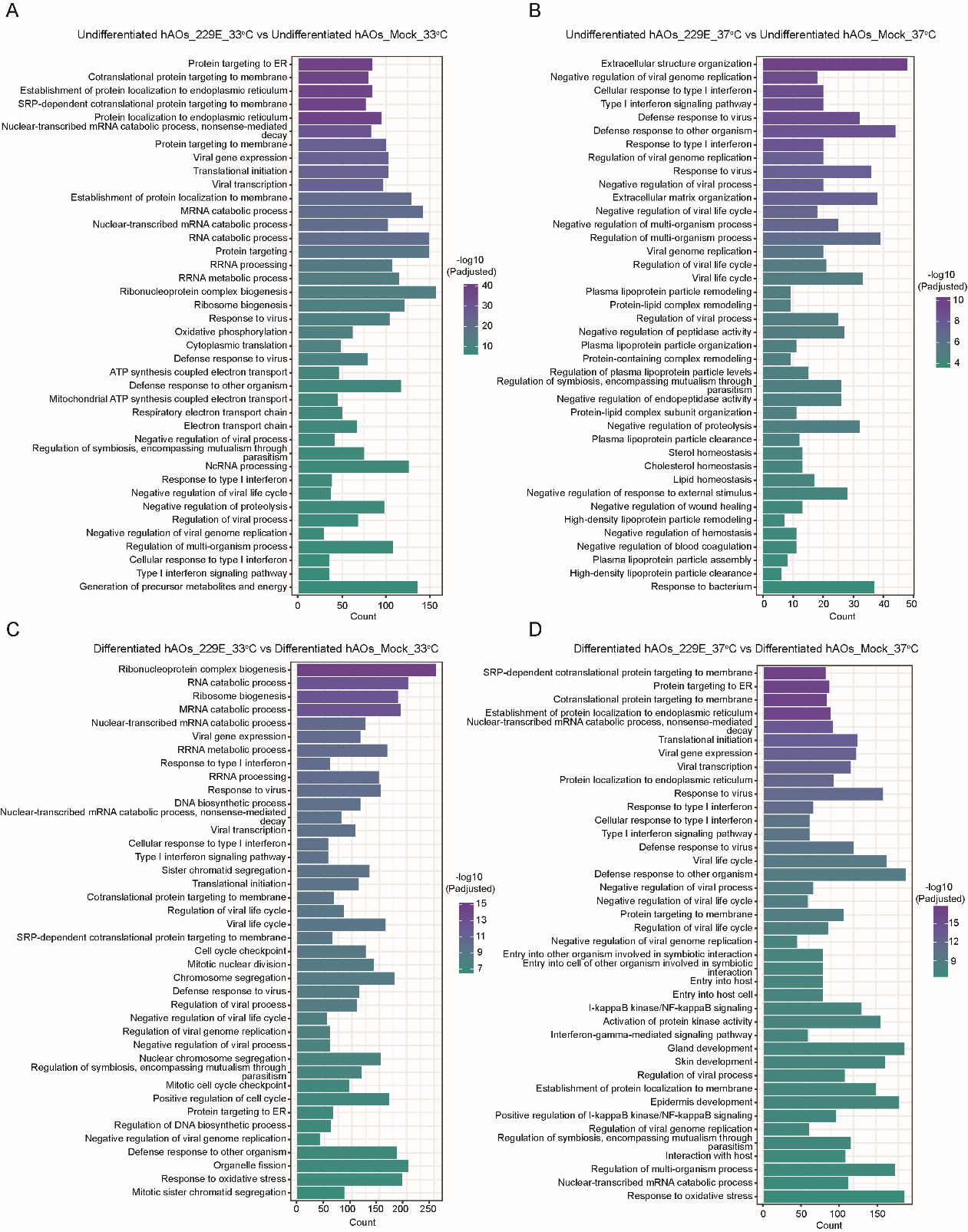


Figure S10. Top 40 significantly enriched pathways by gene ontology (GO) analysis in undifferentiated and differentiated hAOs upon 229E infection that cultured at either 33^o^C or 37^o^C. (A) Undifferentiated hAOs cultured at 33^o^C. (B) Undifferentiated hAOs cultured at 37^o^C. (C) Differentiated hAOs cultured at 33^o^C. (D) Differentiated hAOs cultured at 37^o^C.

Figure S11.


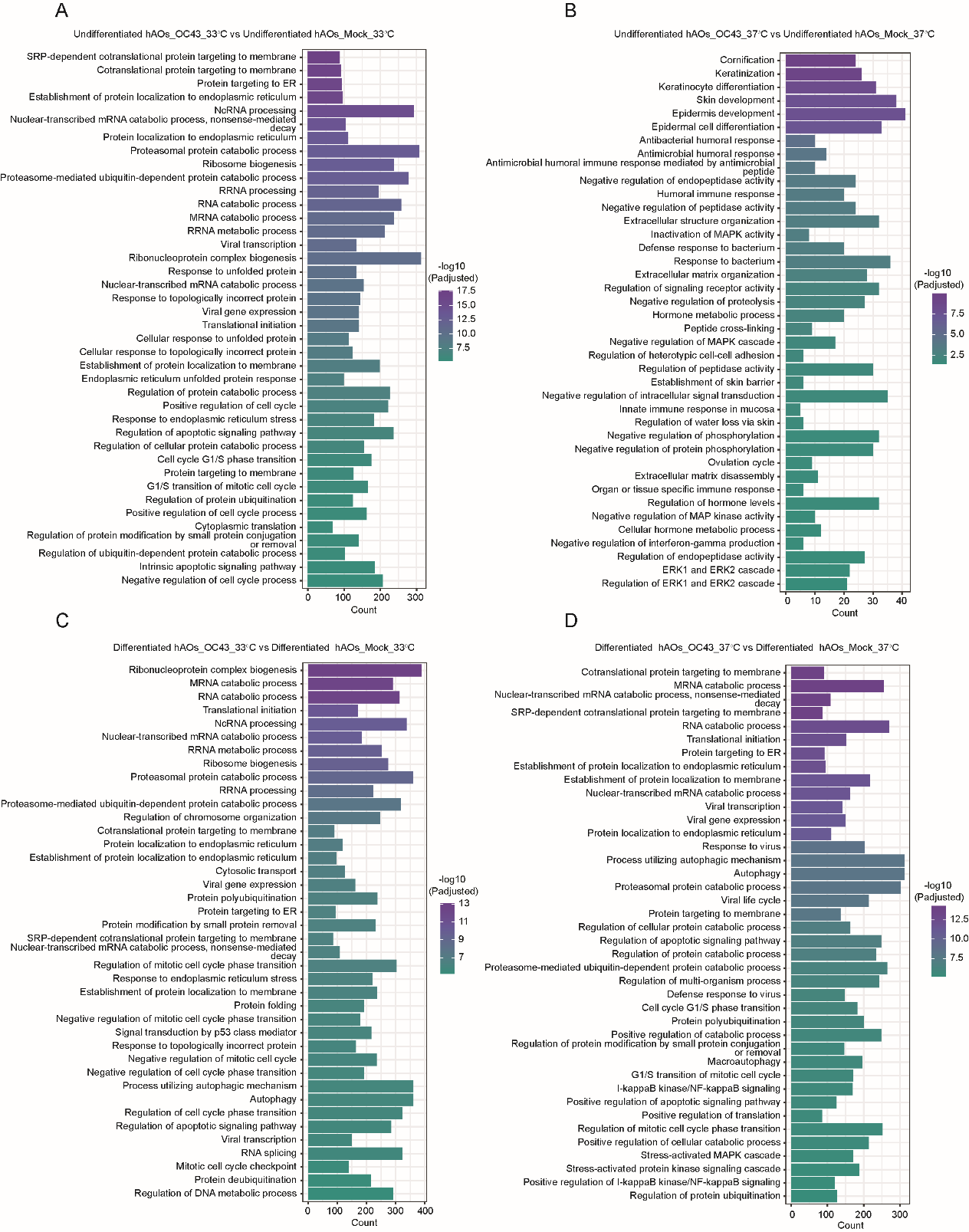


Figure S11. Top 40 significantly enriched pathways by gene ontology (GO) analysis in undifferentiated and differentiated phenotype hAOs upon OC43 infection that cultured at either 33^o^C or 37^o^C. (A) Undifferentiated hAOs cultured at 33^o^C. (B) Undifferentiated hAOs cultured at 37^o^C. (C) Differentiated hAOs cultured at 33^o^C. (D) Differentiated hAOs cultured at 37^o^C.

Figure S12.


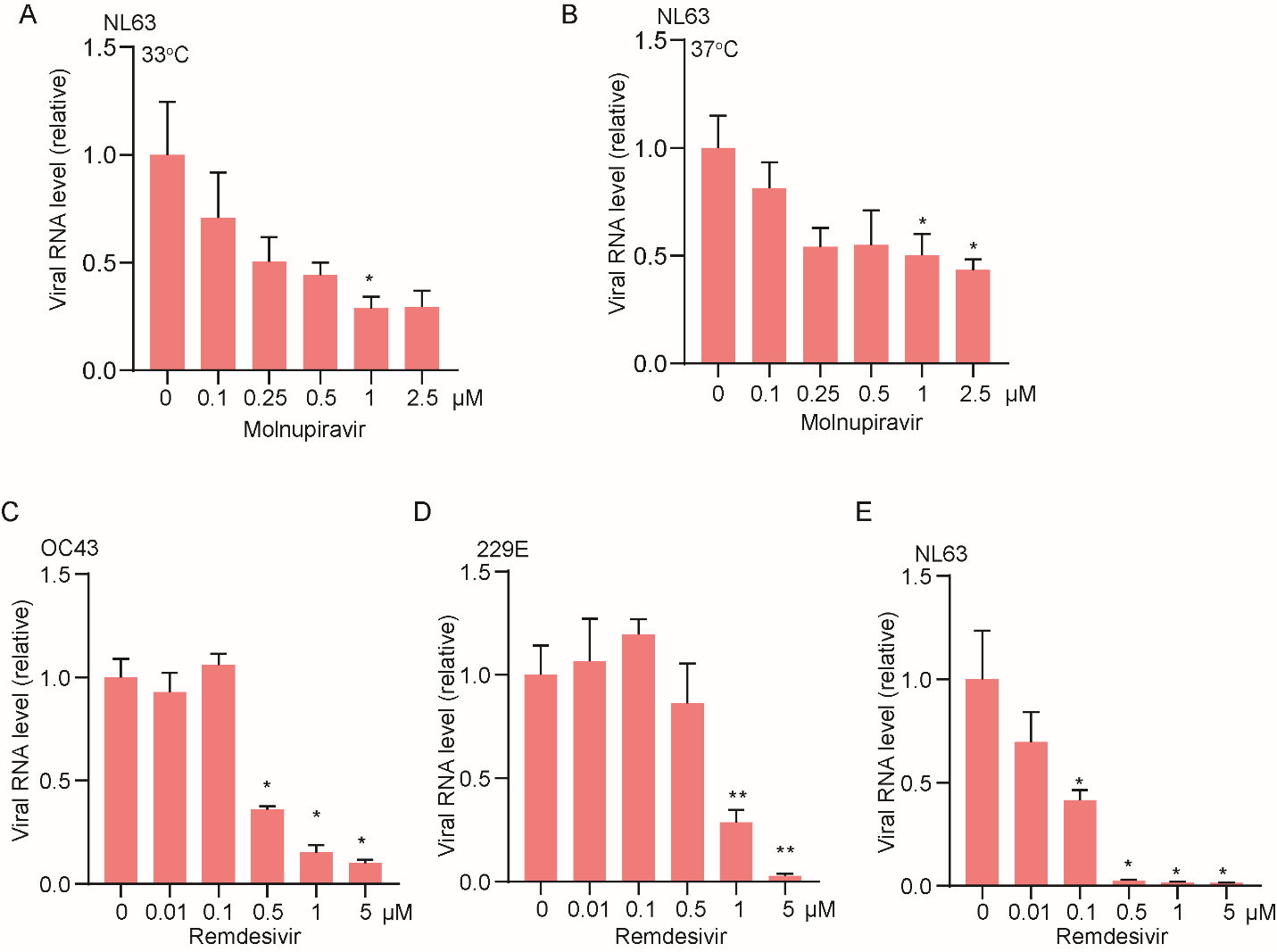


Figure S12. Antiviral treatment against seasonal coronaviruses. (A and B) The inhibitory effect of molnupiravir on NL63 replication in hAOs cultured at 33^o^C and 37^o^C. (C to D) The inhibitory effect of remdesivir on OC43 (C), 229E (D) and NL63 (E) replication in hAOs cultured at 33°C (mean ± SEM, n= 4-6). *P < 0.05; **P < 0.01.

Figure S13


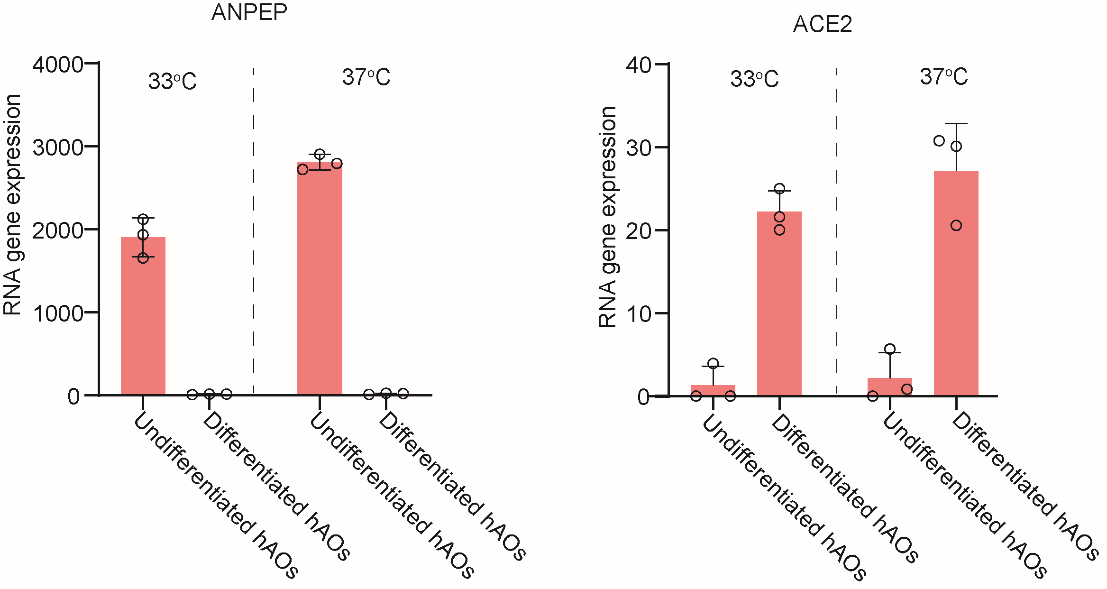


Figure S13. ANEP and ACE2 gene expression in undifferentiated and differentiated hAOs cultured at 33^o^C and 37^o^C.

Table S1. Primers used in this study.

| Gene name | Sequence |
| --- | --- |
| 229E standard plasmid-F | ATGGCTACAGTCAAATGGGC |
| 229E standard plasmid-R | AAGAAGCAGGACTCTGATTACG |
| OC43 standard plasmid-F | GGGAAAGGAGTTTGAGTTTGTAG |
| OC43 standard plasmid-R | CTCGTCAGGATTCCCAGATAA |
| NL63 standard plasmid-F | ACGCAATGCCACTGTTGTTA |
| NL63 standard plasmid-R | GACAACACCGTCATCAGAGA |
| 229E-F | GTCGTCAGGGTAGAATACCTTA |
| 229E-R | CCCGTTTGCGCTTTCTAGT |
| OC43-F | AGCAACCAGGCTGATGTCAATACC |
| OC43-R | AGCAGACCTTCCTGAGCCTTCAAT |
| NL63-F | CTTCTGGTGACGCTAGTACAGCTTAT |
| NL63-R | AGACGTCGTTGTAGATCCCTAACAT |
| Gapdh-F | GTCTCCTCTGACTTCAACAGCG |
| Gapdh-R | ACCACCCTGTTGCTGTAGTAGCCAA |
